# The cochaperone CHIP marks Hsp70- and Hsp90-bound substrates for degradation through a very flexible mechanism

**DOI:** 10.1038/s41598-019-41060-0

**Published:** 2019-03-25

**Authors:** Lucía Quintana-Gallardo, Jaime Martín-Benito, Miguel Marcilla, Guadalupe Espadas, Eduard Sabidó, José María Valpuesta

**Affiliations:** 1 Centro Nacional de Biotecnología (CNB-CSIC), Darwin 3, 28049 Madrid, Spain; 2 Proteomics Unit, Centre de Regulació Genòmica (CRG), Barcelona Institute of Science and Technology (BIST), Barcelona, Spain; 3 Proteomics Unit, Universitat Pompeu Fabra, Barcelona, Spain

## Abstract

Some molecular chaperones are involved not only in assisting the folding of proteins but also, given appropriate conditions, in their degradation. This is the case for Hsp70 and Hsp90 which, in concert with the cochaperone CHIP, direct their bound substrate to degradation through ubiquitination. We generated complexes between the chaperones (Hsp70 or Hsp90), the cochaperone CHIP and, as substrate, a p53 variant containing the GST protein (p53-TMGST). Both ternary complexes (Hsp70:p53-TMGST:CHIP and Hsp90:p53-TMGST:CHIP) ubiquitinated the substrate at a higher efficiency than in the absence of the chaperones. The 3D structures of the two complexes, obtained using a combination of cryoelectron microscopy and crosslinking mass spectrometry, showed the substrate located between the chaperone and the cochaperone, suggesting a ubiquitination mechanism in which the chaperone-bound substrate is presented to CHIP. These complexes are inherently flexible, which is important for the ubiquitination process.

## Introduction

Protein homeostasis, or proteostasis, is the delicate balance of synthesis, folding, trafficking, and degradation of intra- and extracellular proteins. Interruption of this process contributes to a variety of pathologies including neurodegenerative disorders and cancer^1^. Molecular chaperones are central to proteostasis, as they are involved not only in protein folding but also in degradation^2,3^. The fate of the chaperone-bound client protein is dictated by interactions with cochaperones, which drive the substrate towards folding or degradation.

Degradation operates via one of the two general protein degradation pathways: the ubiquitin–proteasome system (UPS), or autophagy. Chaperone-assisted UPS involves interaction of the chaperone-bound substrate with a cochaperone, which acts as an E3 ligase and assists substrate ubiquitination prior to its delivery to the proteasome^4^. The cochaperone C terminus of Hsc70-interacting protein (CHIP) is the best-characterised member of this type of E3 ligases^5,6^. CHIP has three domains^7^ (Supplementary Fig. 1a): the TPR domain which interacts with chaperones Hsp70 and Hsp90; a coiled-coil domain involved in CHIP dimerization; and a U-box domain. It is the latter which connects to an E2 ubiquitin-conjugating enzyme, which in turn ubiquitinates Hsp70- and Hsp90-bound client proteins and targets them to the proteasome for degradation^8^. Although it acts as a homodimer, CHIP has an unusual asymmetric structure with a triangular, concave shape in which one U-box domain is blocked, which might have biological consequences^7^.

Hsp70 is a universally conserved chaperone with important functions in protein folding and disaggregation^9^, but whose pivotal role in protein degradation is only now beginning to be understood. It is a small, two-domain ATPase (70 kDa) (Supplementary Fig. 1b); its substrate-binding domain (Hsp70_SBD_; 25 kDa) recognizes the client protein and acts like forceps to trap it, and the nucleotide-binding domain (Hsp70_NBD_; 45 kDa) controls opening and closing of the Hsp70_SBD_. The two domains are connected by a very flexible, well-conserved linker.

CHIP-mediated degradation in Hsp70 has been characterised in detail^10^. Several studies have shown that in stressful conditions, CHIP overexpression correlates directly with proteasomal degradation of substrates such as cystic fibrosis transmembrane conductance regulator (CFTR)^11^, glucocorticoid receptor (GR)^12^, the E2A transcription factor^13^, tau^14^, huntingtin and ataxin^15^, telomerase^16^, apoptosis signal-regulating kinase 1 (ASK1)^17^, phosphatase and tensin homologue (PTEN)^18^ and p53^6^. Once the Hsp70:substrate complex is formed, it must interact with CHIP for substrate ubiquitination. After this process, the Hsp70:substrate binds cochaperone Bag1, which carries a Bag domain that interacts with Hsp70 and a Ubl domain that links the complex to the proteasome^19^.

Hsp90 is also a very abundant, well-conserved chaperone. It is found as a dimer, with each monomer composed of three domains (Supplementary Fig. 1c): The N-terminal domain (Hsp90_NTD_) bearing an ATP-binding site; the middle domain (Hsp90_MD_) mainly responsible for substrate:protein interactions; and the C-terminal domain (Hsp90_CTD_), bearing the dimerization site^20^. Hsp90 usually stabilises and promotes correct folding of its client proteins with the help of chaperones such as Hsp70 and Hsp40, which initially recognise substrates and transfer them to Hsp90 in a process facilitated by the cochaperone Hop^21^. However, in response to certain stimuli or in the presence of specific inhibitors such as geldanamycin (GA), Hsp90 can direct its bound clients to degradation through the UPS^22–25^. Addition of CHIP to the Hsp90:substrate complex induces a similar response to GA treatment, promoting proteasomal degradation of the client protein^12,26^, though in this case, no physical linker has been found between the Hsp90:client protein complex and the proteasome.

Despite intensive biochemical characterization of the Hsp70 and Hsp90 protein machinery, little is known in structural terms about their behaviour in protein degradation via the ubiquitin–proteasome pathway. Here we generated several of the Hsp70- and Hsp90-based complexes that form during such degradation, and determined their 3D structure using a combination of electron microscopy (EM), image processing, and crosslinking mass spectrometry (XL-MS). Our results show that all of these complexes are extremely flexible, and this dynamic behaviour is necessary not only for interaction between chaperones and a large number of client proteins of all sizes, but also for interaction of the chaperone:substrate complex with the cochaperone CHIP and subsequent substrate ubiquitination.

## Results

### Structure of the Hsp70:substrate:CHIP complex

To characterize the Hsp70- and CHIP-mediated chaperone-dependent ubiquitin proteasome process, we isolated the complex containing chaperone bound to its substrate, in this case p53, a well-described client protein for Hsp70 degradation^27^. We used the monomeric form of p53 (p53-TM), as the tetramer would have added variability and heterogeneity to the structural analysis. The p53-TM expression plasmid, which lacks the oligomerization domain (residues 326–356 (Supplementary Fig. 1d), was a kind gift of Dr Antonio García-Trinidad (Cancer Research UK, Beatson Institute, Glasgow, Scotland). This plasmid has the glutathione S-transferase (GST) gene fused to its N-terminus to increase protein production and stability. Removal of GST after purification nonetheless resulted in rapid p53-TM degradation. We retained the GST molecule and, after confirming that GST does not interact with Hsp70 on its own (Supplementary Fig. 2a), worked with the chimeric protein. The resulting p53-TMGST is a 594-residue protein with two structured domains [the p53 DBD domain (p53_DBD_) and GST], as well as two disordered regions, one (TAD and PRD; 102 residues) that connects GST and p53_DBD_, and the other at the C-terminal part of p53_DBD_ (CT; 76 residues) (Supplementary Fig. 1e).

**Figure 1:**
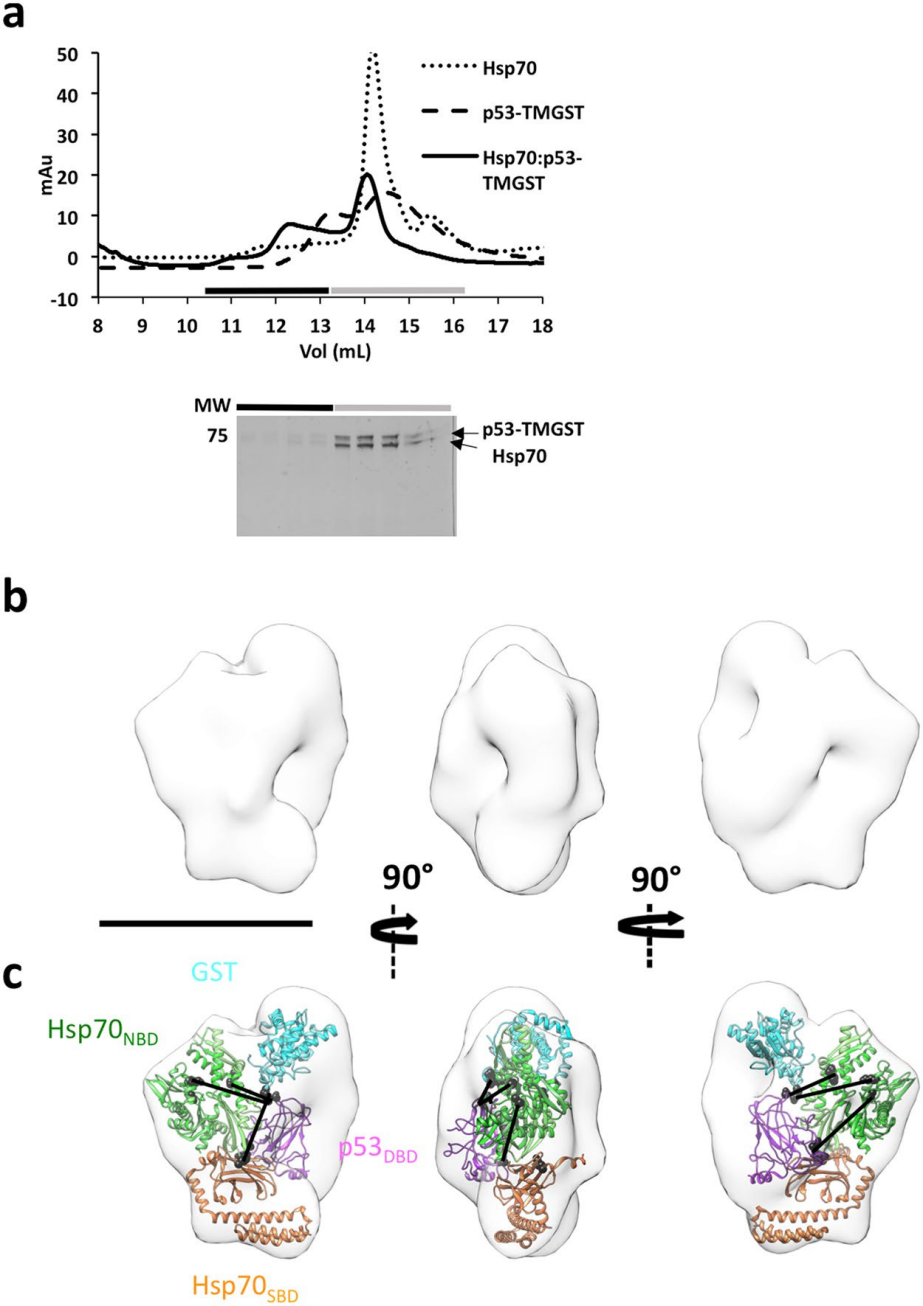
Isolation and structural characterisation of the Hsp70:p53-TMGST complex. (**a**) Top, size-exclusion profile of the complex (continuous line) compared with the profiles for Hsp70 (dotted line) and p53-TMGST (broken line). Bottom, the selected fractions were analysed by SDS-PAGE and stained with Coomassie blue. (**b**) Three orthogonal views of the Hsp70:p53-TMGST complex 3D reconstruction. (**c**) The same views with docking of the chaperone Hsp70 (Hsp70_NBD_ (pdb 5aqz; green) and Hsp70 _SBD_ (pdb 4po2; orange)) and the substrate p53-TMGST (p53_DBD_ (pdb 2ocj; pink) and GST (pdb 1m99; cian)). The DTSSP crosslinks in the images are described in Supplementary Fig. 2c. Only crosslinks between residues located in regions with known atomic structure are depicted in the figure. Bar, 100 Å for (**b**,**c**).

Hsp70 was mixed with p53-TMGST to form the Hsp70:p53-TMGST complex (130 kDa), which could be purified by gel filtration, with fractions containing the two proteins in equimolar amounts (Fig. 1a). This complex was unstable, however, and electron microscopy of the sample had considerable heterogeneity due to free Hsp70 and p53-TMGST (data not shown). We stabilised the complex using GraFix, a gradient technique that allows mild fixation of the proteins in the complex and at the same time serves as an extra purification step for the different proteins and complexes present in the preparation (Supplementary Fig. 2b)^28^. This latter process allowed us to remove the unbound Hsp70 and p53-TMGST. We analysed the stabilised complex by electron microscopy using negative staining and found that despite stabilisation, the Hsp70:p53-TMGST complex still showed appreciable structural heterogeneity.

Given the difficulty of handling this complex and its low molecular mass, we used negative staining to carry out a 3D reconstruction of the Hsp70:p53-TMGST complex. A total of 303 images were acquired using a JEOL JEM1010, and 52,000 particles were selected by an automatic procedure (Xmipp-autopicking^29^) and 2D-classified using Relion^30^. After 3D classification, 18,552 particles forming a majority class were used for a final 3D reconstruction (see Methods). The volume obtained (Fig. 1b; 26 Å resolution; Supplementary Fig. 3) was bulky, with some conspicuous structural features that allowed a preliminary manual docking of the four Hsp70:p53-TMGST complex domains whose atomic structures are known (Hsp70_NBD_, Hsp70_SBD_, p53_DBD_ and GST), followed by a more precise one using Chimera (Fig. 1c). Finally, docking of the different domains was also modified with the help of Crosslinking-Mass Spectrometry (XL-MS) data. The Hsp70:p53-TMGST complex was treated with 3,3′-dithiobis(sulfosuccinimidyl propionate) (DTSSP), which crosslinks adjacent (12 Å) to Lys and Ser residues. The crosslinked complex was trypsin-digested and the resulting peptides analysed by liquid chromatography coupled to tandem mass spectrometry (LC-MS/MS). The crosslinked peptides were identified using the SIM tool^31^, and linked peptides with a score > 4.5 were selected. Seven DTSSP high-quality crosslinks were detected (Supplementary Fig. 2c): two between Hsp70_NBD_ and the p53_DBD_ domain, four between Hsp70_NBD_ and the C-terminal disordered domain of the p53 variant, and one between Hsp70_SBD_ and GST.

These data permitted docking of Hsp70_NBD_ at a central position in the Hsp70:p53-TMGST complex (Fig. 1c), in the vicinity of Hsp70_SBD_ and of the two p53-TMGST domains. The substrate-binding Hsp70_SBD_ was near its interacting partner p53_DBD_, and GST was located distant from the Hsp70_NBD_-Hsp70_SBD_-p53_DBD_ core. The structural heterogeneity observed during the classification procedure indicated a very flexible complex between Hsp70 and the substrate, plausible given the promiscuity of Hsp70, which interacts with clients of a wide array of sizes and structures^32^.

Once the Hsp70:substrate complex forms, the cochaperone CHIP binds it and drives it toward the UPS pathway. To better define the interaction between Hsp70 and CHIP before analysing the ternary complex, we first generated and characterised the Hsp70:CHIP complex. This complex was formed and analysed by size-exclusion chromatography (Supplementary Fig. 4a). Compared to the gel filtration profiles of the individual components, the complex showed a large peak corresponding to free Hsp70 and a smaller, higher mobility peak that contained the two proteins in equimolar amounts, consistent with formation of a binary complex.

**Figure 2:**
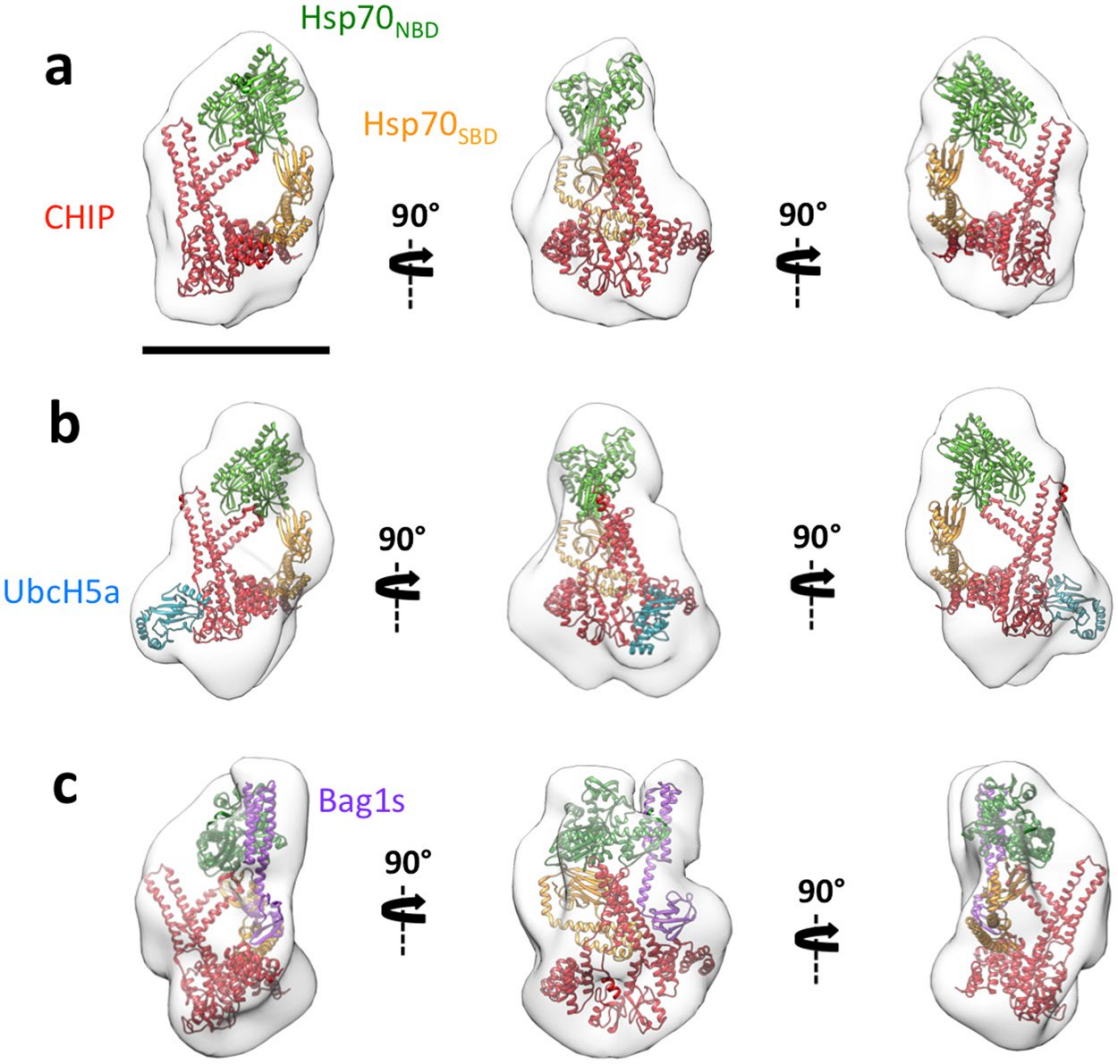
3D reconstructions of Hsp70:CHIP-based complexes. The same three orthogonal views of (**a**) 3D reconstruction of the Hsp70:CHIP complex, with docking of Hsp70 (Hsp70_NBD_, green and Hsp70_SBD_, orange) and CHIP (pdb 2c2l; red), (**b**) 3D reconstruction of the Hsp70:CHIP:UbcH5a complex, with docking of Hsp70, CHIP and UbCH5a (pdb 2oxq; dark blue), (**c**) 3D reconstruction of the Hsp70:CHIP:Bag1s complex, with docking of Hsp70 and Bag1s (from the atomic structure of the Hsp70_NBD_:Bag1 complex, pdb: 4hwi; pink), and CHIP. Bar, 100 Å.

The Hsp70:CHIP complex (140 kDa) was then subjected to GraFix treatment (Supplementary Fig. 3b) and examined by EM. Given the heterogeneity of the sample, we again resorted to a GraFix treatment, which allowed isolation of Hsp70:CHIP (Supplementary Fig. 3b). We negatively stained the Hsp70:CHIP-containing gradient fractions, and chose purest one to record 108 images, from which 92,895 particles were selected. After several 3D classifications, which continued to show considerable Hsp70:CHIP heterogeneity, particles from the majority class (10,805) were selected for 3D refinement (Fig. 2a). The volume obtained was compact and clearly showed the triangular, asymmetric structure of the CHIP dimer. The Hsp70_NBD_ could be placed at the top back of the 3D reconstruction, and the Hsp70_SBD_ below the Hsp70_NBD_, with its C terminus bearing the TPR-binding motif (EEVD) pointing toward the TPR domain of one CHIP monomer (Fig. 2a)^10^. Given the low resolution of the reconstruction (22 Å) (Supplementary Fig. 3), we decided to include an additional protein in the complex to label certain domains and further confirm the docking.

To localise CHIP, we used the E2 ubiquitin-conjugating enzyme UbcH5a (16 kDa; Supplementary Fig. 1f), a member of the UBC4/UBC5 E2 enzyme family^33^, which binds specifically to the accessible U-box of one CHIP monomer^33^. We cloned, expressed and purified UbcH5a and incubated it with Hsp70:CHIP, purifying the resultant Hsp70:CHIP:UbcH5a complex (156 kDa) by gel filtration (Supplementary Fig. 4c) and treating with GraFix (Supplementary Fig. 4d). Final fractions were analysed by negative staining EM, with the purest one used for 3D reconstruction of the complex. 59,245 particles were selected from 178 images, and after several rounds of 2D and 3D classification, 5,158 particles were selected for the final 3D refinement. A volume similar to that of Hsp70:CHIP was generated, with an additional small protrusion at the CHIP site, where the U-box domain is located (UbcH5a binding site^34^) (Fig. 2b). The UbcH5a atomic structure in complex with the CHIP U-box domain (pdb 2oxq^34^) docked well, confirming the CHIP dimer location as the triangular structure seen in our Hsp70:CHIP 3D reconstruction (Fig. 2b).

To confirm Hsp70’s location in the docking, we used Bcl-2-associated athanogene 1 (Bag1), a cochaperone that interacts with the Hsp70_NBD_ and acts as a nucleotide exchange factor that facilitates Hsp70’s ATPase activity^35^. We cloned, expressed, and purified the short isoform of Bag1 (Bag1s; 37 kDa; Supplementary Fig. 1g) and used it to form a complex with Hsp70:CHIP. After gel filtration (177 kDa; Supplementary Fig. 4e) and GraFix treatment (Supplementary Fig. 4f), fractions containing Hsp70:CHIP:Bag1s were analysed by negative staining EM. The most suitable fraction was used for 3D reconstruction of the complex. In all, 78,582 particles were selected from 200 images, and after several rounds of 3D classification, 11,480 particles were selected for final 3D refinement, resulting in a 25 Å resolution model (Fig. 2c). The volume, similar to that of the Hsp70:CHIP complex, again showed the CHIP dimer triangular structure as well as a rod-like protrusion at the top back of the 3D reconstruction (center image in Fig. 2c), where the Bag domain of Bag1s is located^35^. This is the Hsp70-binding domain, which forms a three-helix bundle that contacts the Hsp70_NBD_ subdomains IB and IIB. For docking in the Hsp70:CHIP:Bag1s 3D reconstruction, we used the atomic structures of the human Hsp70_NBD_ and Bag1 from *Arabidopsis thaliana*^19^ (pdb 4hwi), which not only has a high homology with respect to its human counterpart but also contains the Bag and Ubl domains (Fig. 2c). Docking corroborated localisation of the Hsp70_NBD_ to the top of the CHIP structure, with space for the Hsp70_SBD_ at its bottom, and its C terminus positioned toward the TPR domain of the nearest CHIP monomer. We noted that in all three complexes examined, the CHIP dimer interacted with Hsp70 through its concave face.

After determining the structure of the Hsp70:p53-TMGST and Hsp70:CHIP complexes, both of which showed considerable flexibility, we generated the Hsp70:p53-TMGST:CHIP ternary complex, the next step in the Hsp70-dependent ubiquitin/proteasome degradation pathway.

**Figure 3:**
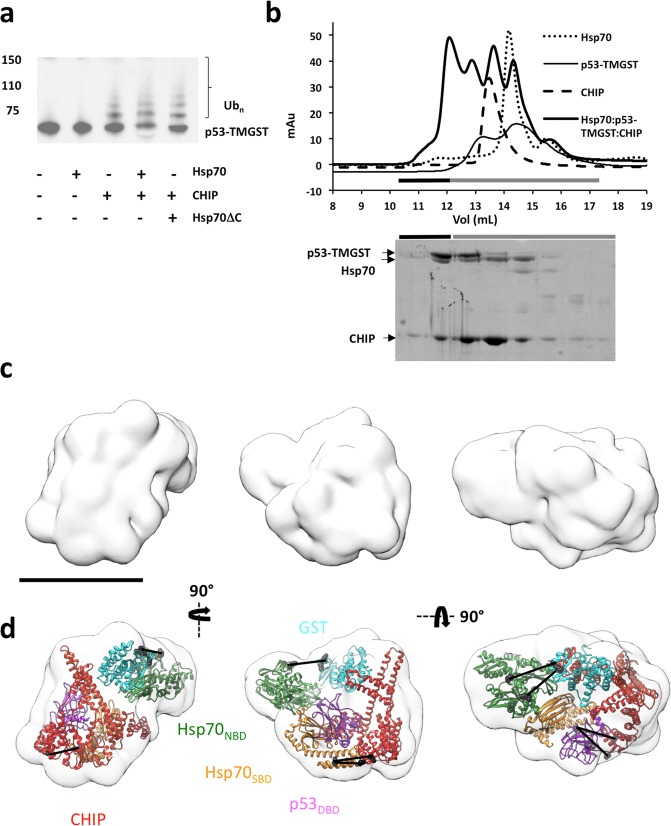
Isolation, ubiquitination activity, and structural characterisation of the Hsp70:p53-TMGST:CHIP complex. (**a**) p53-TMGST ubiquitination assay mediated by Hsp70 and CHIP. For the western blot, an anti-p53 antibody was used. (**b**) Formation of the Hsp70:p53-TMGST:CHIP complex. Top, size-exclusion profile of the complex (thick continuous line) compared with the profiles for Hsp70 (dotted line), p53-TMGST (thin continuous line), and CHIP (broken line). Bottom, the selected fractions were analysed by SDS-PAGE and stained with Coomassie Blue. (**c**) Three orthogonal views of the Hsp70:p53-TMGST:CHIP complex 3D reconstruction. (**d**) The same views with docking of Hsp70 (Hsp70_NBD_ and Hsp70 _SBD_), p53-TMGST (p53_DBD_ and GST), and CHIP. The DTSSP crosslinks in the images are described in Supplementary Fig. 5c. Only crosslinks between residues located in regions with known atomic structure are depicted in the figure. Colours as in Fig. 2. Bar, 100 Å for (**c**,**d**).

To analyse the substrate ubiquitination activity, we incubated p53-TMGST with UbcH5a, E1 (a ubiquitin-activating enzyme), ubiquitin, ATP and MgCl_2_, and ubiquitination activity was analysed alone or with CHIP and/or Hsp70 (Fig. 3a). No substrate was ubiquitinated in the absence of CHIP, and although a lack of Hsp70 did not prevent substrate ubiquitination, its presence increased efficiency. Thus, the chaperone/cochaperone interaction is of value for correct substrate ubiquitination. We confirmed this using an Hsp70 variant (Hsp70ΔC) lacking the C-terminal region and thus the ability to interact with CHIP; this mutant induced ubiquitination activity similar to that observed in the absence of Hsp70 (Fig. 3a).

After confirming Hsp70 and CHIP substrate ubiquitination, we isolated the Hsp70:p53-TMGST:CHIP complex. The three proteins were mixed in equimolar amounts, followed by gel filtration chromatography (Fig. 3b). The elution profile of the complex differed from those of individual proteins, with an additional higher mobility peak; SDS-PAGE showed the three components in equimolar amounts, which confirmed ternary complex formation. As before, GraFix treatment was used to improve complex stability and purity, thus facilitating 3D reconstruction (Supplementary Fig. 5a). After preliminary analysis by negative staining, the most suitable fraction was used for cryoelectron microscopy (cryoEM). Aliquots were vitrified and the grids loaded into a FEI Titan Krios cryoelectron microscope (300 kV) (Supplementary Fig. 5b). We recorded 1,600 movies using K2 post-GIF and selected 160,000 particles that were processed using 2D and 3D classification techniques, again showing a high degree of heterogeneity. The majority class (25,000 particles) was used to generate a 3D reconstruction of the Hsp70:p53-TMGST:CHIP complex (15 Å resolution) (Fig. 3c and Supplementary Fig. 3), which was larger than those described above (200 kDa). Within the mass of the reconstructed volume, the triangular CHIP dimer structure was conspicuous (Fig. 3c, left), as was the V-shaped Hsp70_NBD_ structure; their atomic structures could be easily fitted in these two parts of the complex.

As the remaining domains could not be localised correctly, we complemented the structural data with XL-MS analysis of the complex. Aliquots of Hsp70:p53-TMGST:CHIP were crosslinked with DTSSP and trypsin-digested, and peptides analysed by LC-MS/MS. We obtained four high-quality hits showing inter-subunit crosslinks (Supplementary Fig. 5c). Hsp70_SBD_ had one crosslink between its β-sandwich subdomain and the TPR domain of one of the CHIP monomers, which left the Hsp70 C-terminal EEVD motif and the CHIP TPR domain in the vicinity the Hsp70-CHIP-interacting regions. One high-quality crosslink was found between the Hsp70_NBD_ and the GST domain. The C-terminal, unstructured part of p53-TMGST had two crosslinks; one with the Hsp70_NBD_ and another with one of the CHIP TPR domains.

All of these DTSSP crosslinks helped us to build a model of the Hsp70:p53-TMGST:CHIP complex (Fig. 3d). The two structured domains of the substrate were found between Hsp70 and CHIP, with the p53_DBD_ near the Hsp70_SBD_ (not surprising, given that these are the Hsp70-p53 binding domains) and the U-box domain of one of the CHIP monomers, and GST near the CHIP central coiled-coil domain. The Hsp70_SBD_ localised near the TPR domain of the CHIP monomer closest to the chaperone, whereas the Hsp70_NBD_ was further from CHIP and the substrate. The long, open structure of the Hsp70:p53-TMGST:CHIP complex resembles the model for the Hsp70:CHIP:UbcH5 complex proposed by Zhang and collaborators^36^.

### Structure of the Hsp90:substrate:CHIP complex

Before generating a complex between Hsp90, the cochaperone CHIP and a substrate, we generated and structurally characterised complexes between Hsp90 and a substrate or CHIP. We used p53-TMGST as a substrate, after confirming that GST alone does not bind to Hsp90 (Supplementary Fig. 6a). Several studies have characterised the interaction between Hsp90 and p53_DBD_^37–41^, some of which have shown that this interaction is dynamic and involves several Hsp90 regions.

**Figure 4:**
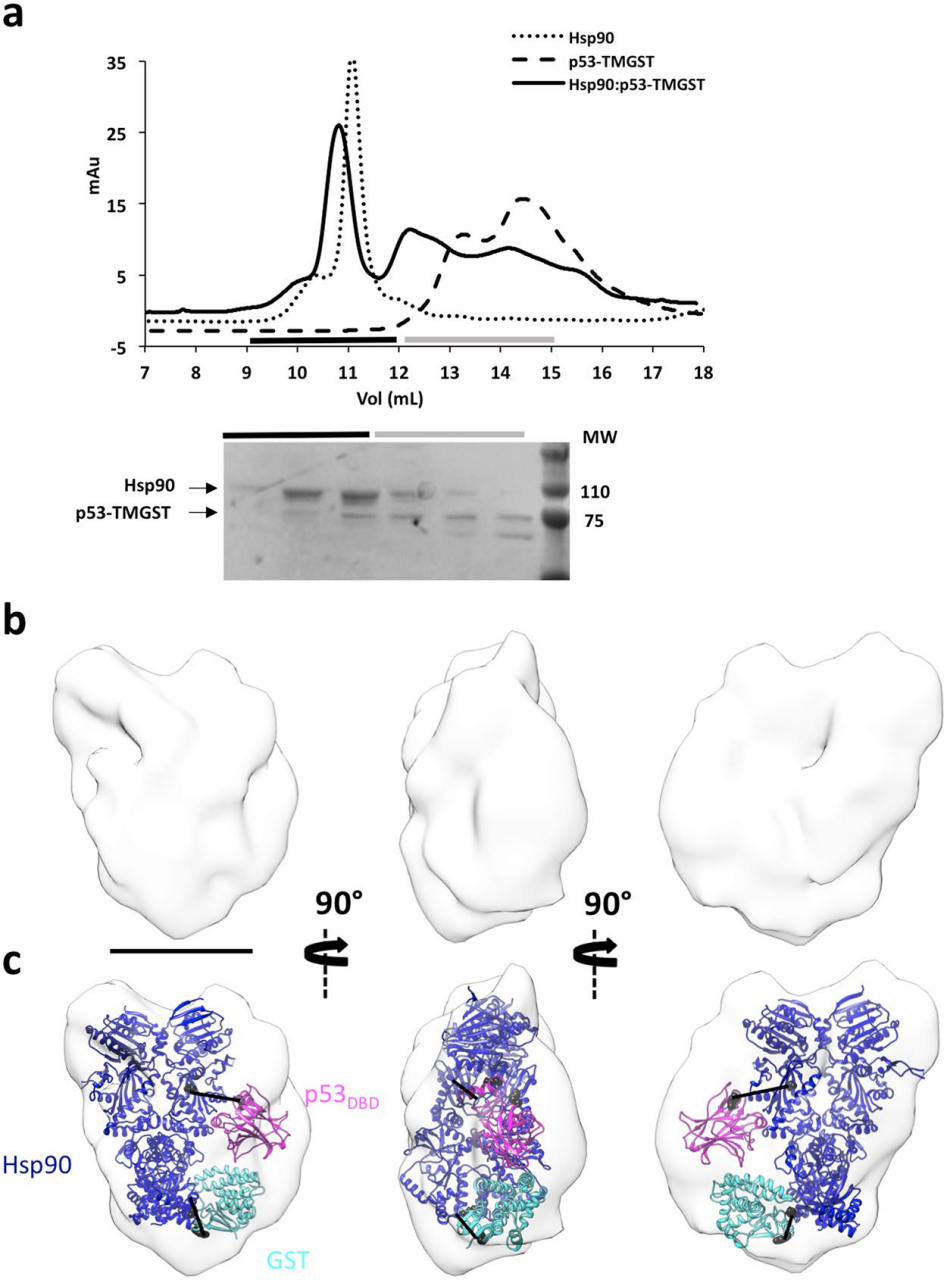
Isolation and structural characterisation of the Hsp90:p53-TMGST complex. (**a**) Top, size-exclusion profile of the complex (continuous line) compared with the profiles for Hsp90 (dotted line) and p53-TMGST (broken line). Bottom, the selected fractions were analysed by SDS-PAGE and stained with Coomassie Blue. (**b**) Three orthogonal views of the Hsp90:p53-TMGST complex 3D reconstruction. (**c**) The same views with docking of Hsp90 (pdb 5fwk; dark blue) and p53-TMGST. The DTSSP crosslinks in the images are described in Supplementary Fig. 6c. Only crosslinks between residues located in regions with known atomic structure are depicted in the figure. Bar, 100 Å for (**b**,**c**).

Hsp90 and p53-TMGST were combined in the presence of ATP, followed by size-exclusion chromatography (Fig. 4a). The elution profile showed a peak with higher mobility than that of the Hsp90 control. The fractions in this peak showed both Hsp90 and p53-TMGST proteins, although not in stoichiometric amounts (see below). As negative staining EM of the fractions containing the two proteins again indicated considerable heterogeneity, GraFix treatment was used to stabilise and further purify the Hsp90:p53-TMGST complex (240 kDa) (Supplementary Fig. 6b). The gradient separated two peaks, one containing dimeric Hsp90 and a second, higher-mobility peak that we analysed by EM. We recorded 241 images, from which 52,067 particles were selected for 2D and 3D classification; the majority class (21,252 particles) was used for 3D refinement. Although Hsp90 is a homodimer that can bind two p53_DBD_ molecules^39^, no symmetry was apparent after several refinement rounds, and we did not apply C2 symmetry in the 3D reconstruction. The asymmetry of the complex, which indicates 1:1 Hsp90 homodimer:p53-TMGST stoichiometry, would explain the lack of equimolarity in the purified Hsp90:p53-TMGST fractions. The final volume (Fig. 4b) (19 Å resolution) (Supplementary Fig. 3) showed clear asymmetry and a shape that rendered straightforward docking of the human Hsp90 closed form (Fig. 4c) (extracted from pdb 5fwk^42^). This Hsp90 docking allowed space for only one p53-TMGST molecule, but the low resolution of the 3D reconstruction did not permit precise p53_DBD_ and GST localisation. Using XL-MS with DTSSP as a crosslinker, we obtained three high-confidence crosslinks (Supplementary Fig. 6c); one between the Hsp90_MD_ and p53_DBD_, two connecting the Hsp90_CTD_ with GST or the p53-TMGST C-terminal unstructured region. These data allowed us to position the p53_DBD_ near the Hsp90_MD_ (Fig. 4c), confirming previous NMR analyses showing this Hsp90 region binding the p53_DBD_^39,41^, whereas GST localised near the Hsp90_CTD_.

**Figure 5:**
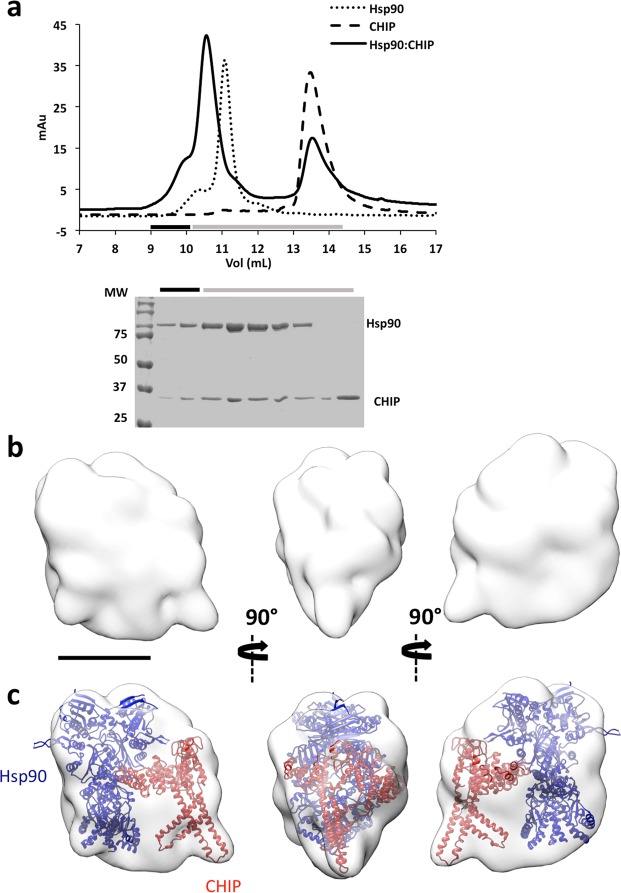
Isolation and structural characterisation of the Hsp90:CHIP complex. (**a**) Top, size-exclusion profile of the complex (continuous line) compared with the profiles for Hsp90 (dotted line) and p53-TMGST (broken line). Bottom, the selected fractions were analysed by SDS-PAGE and stained with Coomassie Blue. (**b**) Three orthogonal views of the Hsp90:p53-TMGST complex 3D reconstruction. (**c**) The same views, with docking of Hsp90 and CHIP. Bar, 100 Å for (**b**,**c**).

To analyse the Hsp90:CHIP complex (250 kDa), we incubated the two proteins in the absence of nucleotide, followed by gel filtration (Fig. 5a). Negative staining EM indicated substantial heterogeneity, which we again attributed to complex flexibility. We therefore stabilised the Hsp90:CHIP complex using GraFix (Supplementary Fig. 6d). The centrifugation profile showed two main peaks, one of lower mass (~200 kDa) assigned to free, dimeric Hsp90 as confirmed by EM, and another of lower mobility (~250 kDa) that contained the Hsp90:CHIP complex. Using the most homogeneous fraction of the latter peak, we recorded 225 images from which we selected 50,698 particles that were classified by 2D and 3D procedures, and the majority class (14,479 particles) was used to generate a final 3D reconstruction (19 Å resolution) (Fig. 5b). The shape of the structure allowed unequivocal docking of both homodimers (Fig. 5c), with Hsp90 in the larger, almond-shaped mass and CHIP in the smaller triangular mass. This docking placed the asymmetrical CHIP structure in contact with the Hsp90_MD_, where most Hsp90 client proteins bind and where the p53_DBD_ was found in the Hsp90:p53-TMGST complex. The Hsp90-CHIP binding domains (TPR domains in the CHIP dimer and CTD domains in the Hsp90 dimer) were located far from one another, but within reach of the long stretch of disordered residues (32) at the end of the structured CTD. In this docking, CHIP interacted with Hsp90 through its concave face, leaving the only E2 binding site available in the CHIP asymmetric structure on the side opposite Hsp90, where the substrate would be located.

**Figure 6:**
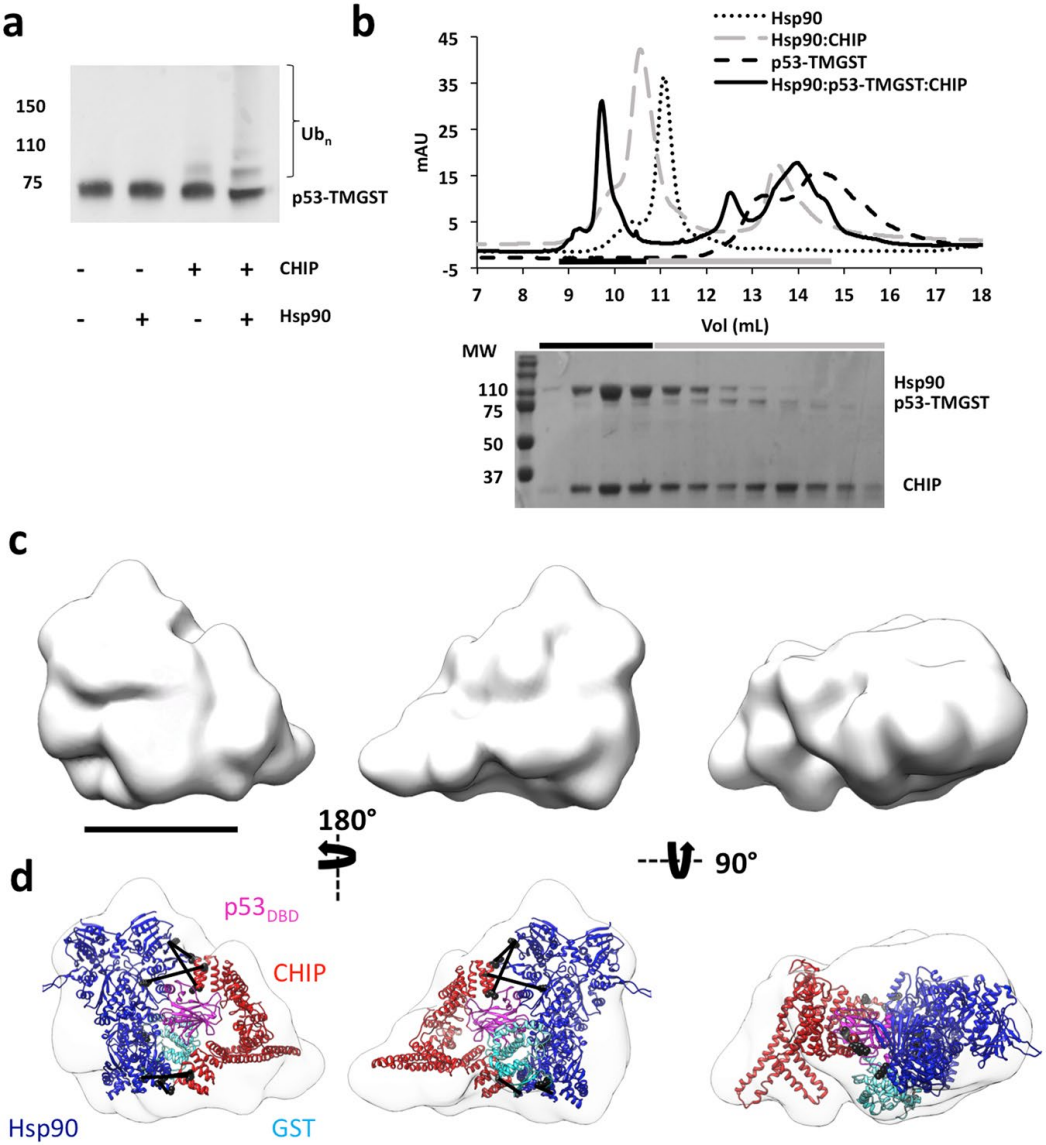
Isolation, ubiquitination activity and structural characterisation of the Hsp90:p53-TMGST:CHIP complex. (**a**) p53-TMGST ubiquitination assay mediated by Hsp90 and CHIP. For the western blot, an anti-p53 antibody was used. (**b**) Formation of the Hsp90:p53-TMGST:CHIP complex. Top, size-exclusion profile of the complex (continuous line) compared with the profiles for Hsp90 (dotted line), p53-TMGST (broken lines), and the Hsp90:CHIP complex (grey broken line). Bottom, the selected fractions were analysed by SDS-PAGE and stained with Coomassie Blue. (**c**) Three orthogonal views of the Hsp90:p53-TMGST:CHIP complex 3D reconstruction. (**d**) The same views with docking of Hsp90, p53-TMGST (p53_DBD_ and GST), and CHIP. The DTSSP and DSSO crosslinks depicted in the images are described in Supplementary Fig. 7c. Only crosslinks between residues located in regions with known atomic structure are depicted in the figure. Not all the crosslinks are depicted in every view. Bar, 100 Å for (**c**,**d**).

We then generated Hsp90:p53-TMGST:CHIP, the next step in the Hsp90-dependent ubiquitin/proteasome degradation pathway. To analyse substrate ubiquitination activity, we incubated p53-TMGST with all the components (UbcH5a, the E1 ubiquitin-activating enzyme, ubiquitin, ATP and MgCl_2_), and ubiquitination activity was tested alone or with Hsp90 and/or CHIP (Fig. 6a). Results showed that, as for Hsp70, the lack of Hsp90 did not prevent substrate ubiquitination, although this was more efficient when Hsp90 was present.

After confirming Hsp90 and CHIP ubiquitination activity, we isolated the Hsp90:p53-TMGST:CHIP complex for structural analysis by incubating the three proteins in equimolar amounts, followed by gel filtration (Fig. 6b). The elution profile of Hsp90:p53-TMGST:CHIP differed from those of the individual components, with an additional higher-mobility peak that contained all three components of the complex, as found by SDS-PAGE. After confirming Hsp90:p53-TMGST:CHIP heterogeneity by negative staining, we stabilised the sample using GraFix (Supplementary Fig. 7a). Electrophoresis analysis of the gradient showed several peaks, the heaviest of which contained the three ternary complex components (~320 kDa). Aliquots of the most suitable fraction were vitrified for cryoEM, the grids loaded into a FEI Talos Arctica cryoelectron microscope (200 kV) (Supplementary Fig. 7b), and 1200 movies were recorded using a Falcon III direct detector. A total of 120,000 particles were selected automatically and processed by 2D and 3D classification. The majority class (10,000 particles) was used for 3D reconstruction of Hsp90:p53-TMGST:CHIP (Fig. 6c).

Given the flexibility of the complex, the resolution was not high (13 Å resolution) (Supplementary Fig. 3); however, the pronounced shape of the Hsp90 and CHIP dimers allowed straightforward docking of the chaperone and cochaperone (Fig. 6d). The longitudinal axes of the two dimers were in an almost orthogonal position, and with the base of the triangular CHIP structure seated on one of the Hsp90 monomers. p53-TMGST was located in the unassigned mass between the chaperone and the cochaperone; to localise its two domains more precisely, we used XL-MS and crosslinked the complex with two different reagents, DTSSP and disuccinimidyl sulfoxide (DSSO). In the first case, two high-quality hits were generated (Supplementary Fig. 7c) which helped locate GST near the Hsp90_CTD_. When DSSO was used, we obtained seven high-quality hits between Hsp90 and CHIP: four hits between one CHIP TPR domain and the Hsp90_CTD_; two hits between the other CHIP TPR domain and the Hsp90_NTD_ (Supplementary Fig. 7c); and one hit between one of the CHIP TPR domains and one Hsp90_MD_ residue. Based on combination of the Hsp90:p53-TMGST:CHIP 3D reconstruction results with the XL-MS data, we concluded that after CHIP binding to Hsp90:p53-TMGST, the two p53-TMGST domains are displaced by CHIP and move slightly from their original position in the complex towards the interior of the cavity formed by chaperone and cochaperone; CHIP again shows its concave face to both the chaperone and the substrate.

### Flexibility of the interaction between CHIP and the chaperones

All the complexes described (chaperone:substrate, chaperone:CHIP, or chaperone:substrate:CHIP) showed a very high degree of flexibility that prevented their structural determination at high resolution (Supplementary Fig. 8). This likely has biological significance, since the chaperones as well as the cochaperone must interact with substrates of different size and shape.

**Figure 7:**
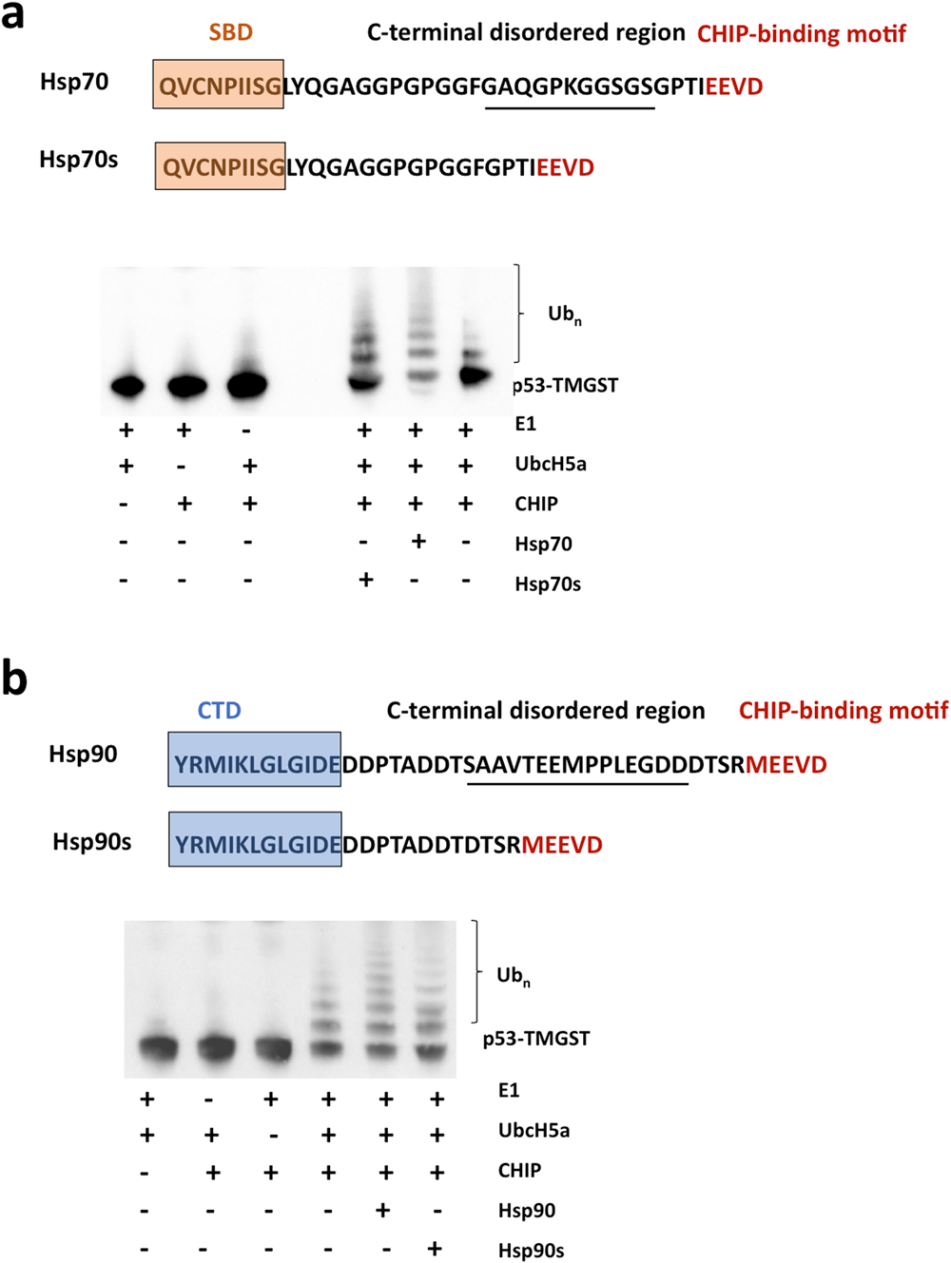
p53-TMGST ubiquitination mediated by chaperones Hsp70, Hsp90, and their variants with shorter, disordered C-terminal sequences. (**a**) Top, sequence of the full-length C-terminal region of Hsp70 and its shorter variant. Bottom, western blot of a ubiquitination experiment using an anti-p53 antibody. (**b**) Top, sequence of the full-length C-terminal region of Hsp90 and its shorter variant. Bottom, western blot of a ubiquitination experiment using an anti-p53 antibody.

To confirm this hypothesis, for chaperone/CHIP interactions, we focused on the long stretch of disordered residues at the C-terminus of Hsp70 and Hsp90 (28 and 27 residues, respectively) immediately before the TPR-binding motif (EEVD and MEEVD, respectively) (Fig. 7). These long stretches make Hsp70:CHIP and Hsp90:CHIP dynamic, tethered complexes in which the chaperone and the cochaperone act independently^43^. To determine the importance of this flexibility for the CHIP-induced ubiquitination of the chaperone:substrate complex, we generated Hsp70 and Hsp90 variants (Hsp70s and Hsp90s, respectively) in which the central residues of the C-terminal disordered regions were removed (11 residues in Hsp70; 15 residues in Hsp90; Fig. 7) The rationale behind this experiment is that decreasing the length of the linker that connects the chaperones and CHIP should make a more rigid complex, which would negatively affect the ubiquitination activity. The substrate ubiquitination assays showed that the shorter Hsp variants had less activity than the wild-type controls (Fig. 7), confirming the importance of these disordered regions and the need for a flexible, tethered chaperone:CHIP complex for efficient ubiquitination of chaperone-bound substrate.

## Discussion

Molecular chaperones, especially Hsp70 and Hsp90, have been shown in recent years to participate not only in protein folding but also in degradation^8,44^, conferring on them an essential role in various aspects of proteostasis. In these two processes, Hsp70 and Hsp90 interact with a wide array of proteins through a poorly characterised recognition mechanism involving weak interactions, generating chaperone:substrate complexes that are intrinsically very flexible^32^. We observed this flexibility through EM analysis of the structure of Hsp70 and Hsp90 in complex with substrate and/or cochaperone; this was only mitigated by mild crosslinking. This flexibility (Figs 1 and 4) is likely very important for allowing these two chaperones to accommodate many different substrates. Similar flexibility was observed when CHIP interacted with Hsp70 or Hsp90, again reduced only in part by crosslinking, which promoted a more compact conformation (Figs 2 and 5, respectively). Subsequent interaction of CHIP with Hsp70:p53-TMGST or Hsp90:p53-TMGST also gave rise to flexible complexes (Figs 3 and 6, respectively) that direct the bound substrate toward the UPS pathway. CHIP interacts with these chaperones through the TPR domain of one of its monomers and a specific C-terminal chaperone motif (EEVD in Hsp70; MEEVD in Hsp90)^45^. Because the regions that precede these motifs in both chaperones are long and unstructured, the chaperone:cochaperone complexes formed are also very flexible.

Structural analysis of these complexes cannot be performed by X-ray crystallography (because of their flexibility) nor by NMR (because of their large molecular mass). Electron microscopy combined with image processing techniques is the only approach able to provide useful structural information, and use of sophisticated classification techniques to manage large amounts of heterogeneous data allows a broader view of the distinct conformations of these complexes. Nonetheless, the flexibility of the complexes analysed here was such that no classification analysis was possible until mild fixation was applied to reduce the number of conformations (without affecting complex structure). This treatment was sufficiently successful for all the analysed complexes to allow detection of majority populations adequate for 3D reconstruction. By combining these results with XL-MS^46,47^, we generated pseudoatomic models of the initial complexes in the Hsp70- and Hsp90-dependent UPS-mediated degradation pathways.

**Figure 8:**
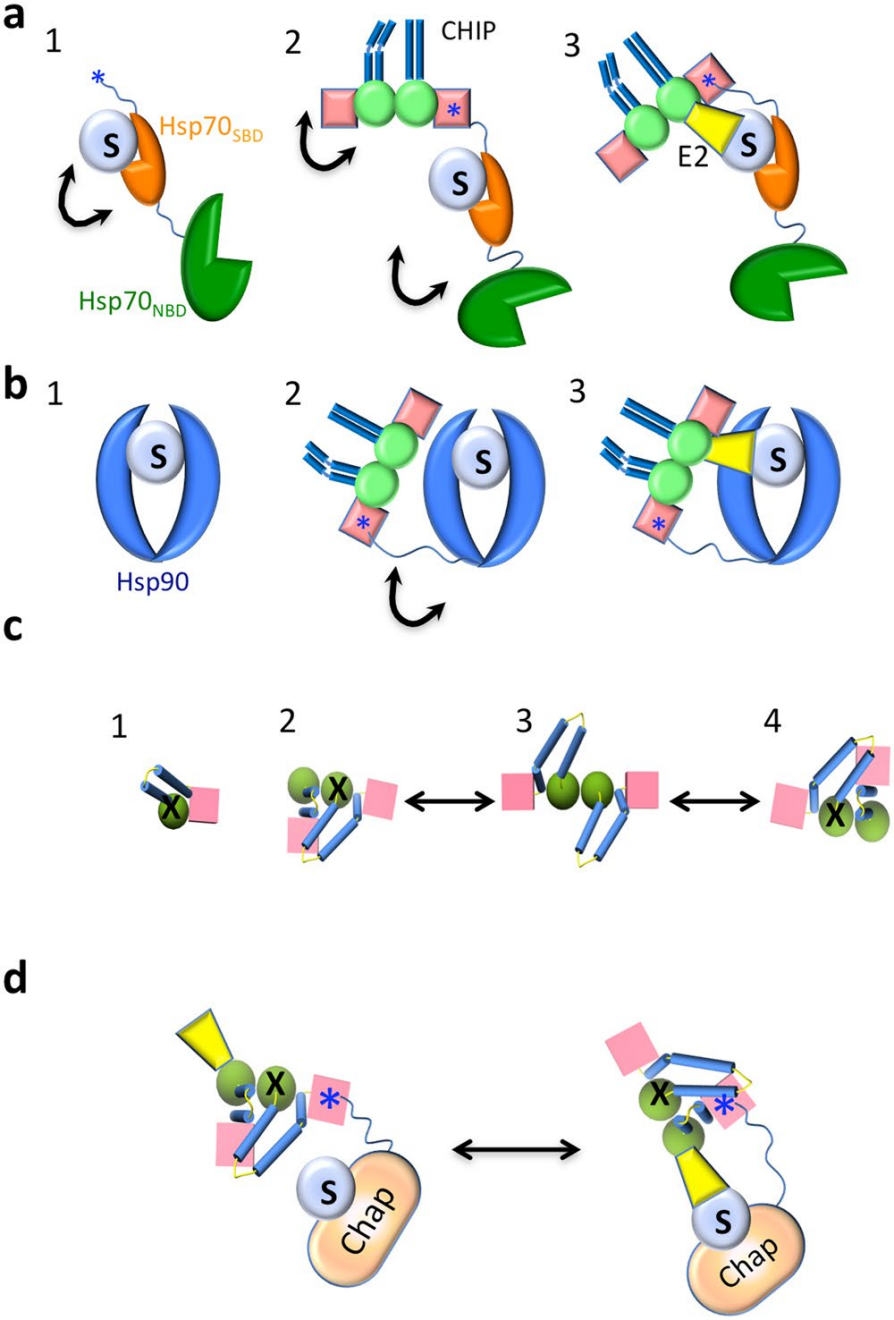
Flexibility in the interaction between chaperone, substrate and CHIP. (**a**) For Hsp70: (1) a flexible complex is formed through weak interactions between the Hsp70_SBD_ and the substrate (S) (black arrows represent flexibility). (2) CHIP binds to Hsp70:S through the Hsp70 IEEVD motif (blue star) at the C-terminus of the chaperone and the TPR domain of one of the CHIP monomers. The EEVD motif is at the end of a long unstructured region (32 residues), which makes the Hsp70-CHIP interaction very flexible. (3) CHIP movements, together with binding of an E2 ubiquitin-conjugating enzyme (i.e., UbCH5a; yellow), allow substrate ubiquitination. (**b**) For Hsp90: (1) a flexible complex is formed through weak interactions between the Hsp90 substrate-binding region and the substrate. (2) CHIP binds to Hsp90:S through the MEEVD motif at the end of the long, unstructured region (32 residues) of one of the Hsp90 monomers and the TPR domain of one of the CHIP monomers, generating a flexible ternary complex with the substrate positioned between Hsp90 and CHIP. (3) CHIP movements, together with binding of an E2 enzyme, allow substrate ubiquitination. (**c**) Model of the conformational changes undergone by CHIP (structure observed from the top). The crystal structure of full-length CHIP is that of an asymmetric dimer^7^. (1) Each monomer is composed of a TPR domain (pink cube), a U-box domain (green sphere), and a dimerization domain (blue cylinders). Molecular dynamics studies^50^ have shown that the monomer in solution is stabilized through TPR-Ubox interactions which block the E2 binding site (X). (2) In the dimer, the two helix-coils come together such that one bends and generates two smaller, kinked helices. The dimer switches between the two possible asymmetric conformations (2 and 4) through a short-lived symmetric conformation (3). Dimer formation activates CHIP by unlocking one of the two E2 binding sites (the one without X). (**d**) Model of chaperone-bound, substrate ubiquitination mediated by CHIP and an E2 enzyme. When interacting with the chaperone:substrate complex, CHIP switches between the two asymmetric conformations described in (**c**), and E2 binding can alternate between the two potential E2-binding sites. Whereas the conformation on the left is that found in the 3D-reconstructed CHIP-based complexes in this study, the only productive interaction is that on the right, as it permits contact between the E2 enzyme and the substrate.

Our findings describe the formation of an initial, very flexible chaperone:substrate complex (Figs 1, 4 and 8a,b) and its subsequent interaction with the E3 ligase CHIP, which positions the substrate between the chaperone and the cochaperone (Figs 2, 5 and 8a,b). The chaperone:cochaperone interaction is very important for more efficient substrate ubiquitination, as the absence of chaperone (or the interaction between the chaperone and CHIP) induces a lower degree of ubiquitination (Figs 3a and 6a). This interaction is also very flexible, with the chaperone:substrate complex and CHIP behaving like “beads on a string”^43^. This plasticity allows the cochaperone to scan a large space in search of productive interactions with a substrate, followed by E2 binding to CHIP, and substrate ubiquitination (Figs 3, 6 and 8a,b). Chaperone:cochaperone flexibility is extremely important, since CHIP clients vary in size and shape, as well as the location of the lysines to be ubiquitinated. When we reduced the length of the unstructured region linking CHIP with the chaperone:substrate complex (Fig. 7), ubiquitination was decreased, which further strengthens this idea.

CHIP in solution is a homodimer and its crystal structure is asymmetric, which causes occlusion of one of the two potential E2 binding sites, a finding supported by calorimetric studies^7^. This unusual structure has been challenged by other structural and biochemical studies that favour a symmetric structure^34,48^. This apparent discrepancy has been explained by a model of conformational flexibility^49^, in which the CHIP dimer switches between two opposed asymmetric conformations (Figs 2, 4 and 8c) through a short-lived, symmetric conformation (Figs 3 and 8c). A recent molecular dynamics simulation with the CHIP monomer and dimer^50^ calculated that a monomer in solution would be most stable when the TPR and U-box domains interact, which would occlude the E2 binding site (Figs 1 and 8c). Such a monomer would thus be non-functional, and CHIP E3 ligase activity could only be activated after dimer formation, when one of the two E2 binding sites would become accessible to the enzyme. Our 3D reconstructions of all the CHIP-based complexes reported here support an asymmetric structure (Figs 2, 3, 5 and 6). In all these cases, however, the accessible E2 binding site was on the side of the CHIP structure opposite the substrate, which would prevent its ubiquitination (Fig. 8d, left). Our results showing that the chaperone:CHIP complexes nonetheless ubiquitinate the substrate clearly support a model of CHIP conformational flexibility that would eventually lead to the opposite asymmetric conformation in which CHIP-bound E2 contacts the substrate and triggers its ubiquitination (Fig. 8d, right).

Here we carried out structural characterisation of the initial chaperone-based complexes formed in the UPS pathway and showed their intrinsic flexibility, which is necessary for recognition and trapping of the substrate as well as its recognition and ubiquitination by the E3 ligase.

## Methods

### Protein expression and purification

Cells containing the pPROEX HTa plasmid with the desired gene for hBag1s, hUbcH5a, hCHIP, (Entrez Gene codes 573, 7321, 10273 respectively); the pETDuet-1 plasmid for hHsp90 (Entrez Gene code 3320); the pQE-30 plasmid for Hsp70 (Entrez Gene code 3303) and the pGEX-4T1 for p53-TMGST, and p53_DBD_ (Entrez Gene code 7157) were cultured on 20 mL LB medium with chloramphenicol and ampicillin (overnight, 37 °C). The cells were used as inoculum for 2 L of LB medium with the appropriate antibiotic and were cultured at 37 °C to A_600_ = 0.5. Recombinant proteins were expressed by adding isopropyl 1-thio-β-D-galactopyranoside (IPTG; 1 mM final concentration). For Hsp90, Hsp40, CHIP, UbcH5a, Bag1s, p53_DBD_ and GST, expression was induced at 30 °C (4 h); Hsp70 and p53-TMGST were expressed at 16 °C (overnight). Cells were then pelleted (5000 × g, 15 min, 4 °C) and resuspended in 30 mL lysis buffer (150 mM KCl, 5 mM MgCl_2_, 5 mM EDTA, 0.5% Triton X-100, 15% glycerol, 5 mM DTT, 1 mM PMSF, 50 mM Hepes pH 8.0) and stored at −20 °C.

Cells were thawed, resuspended and supplemented with a protease inhibitor cocktail (Roche). Lysozyme was added to the lysate (1 mg/mL final concentration) and incubated (30 min, 4 °C, with shaking). Lysates were cleared by centrifugation (20,000 × g, 30 min, 4 °C). The soluble fraction was filtered through 0.22 µm filters (Millipore) and dialyzed using 1 L binding buffer (500 mM KCl, 15% glycerol, 1 mM DTT, 20 mM imidazole, 20 mM Hepes pH 7.4) for 2 h at 4 °C. The sample was loaded onto a Ni-NTA agarose column (HisTrap FF 5 mL, GE Healthcare) using an AKTA prime FPLC (GE Healthcare). The column was washed with 5 column volumes (cv) of binding buffer, followed by the same buffer with 500 mM imidazole (elution buffer) to elute the protein. Fractions were collected and analysed by SDS-PAGE; those containing the protein were pooled and concentrated to 5 mL using different cut-off (depending on protein size) Amicon Ultra 15 filters (Millipore) and loaded onto a HiLoad 16/60 Superdex 200 (GE Healthcare) pre-equilibrated with S buffer (100 mM KCl, 5% glycerol, 1 mM DTT, 20 mM Hepes pH 7.4). Proteins were eluted in 1 cv and fractions were analysed by SDS-PAGE and blue native protocols to confirm oligomerization status^51^. Fractions with the highest purity and correct oligomerization state were pooled and concentrated to 1 mL using an Amicon Ultra 15. Protein concentration was determined by the Bradford method^52^. Glycerol (5% final concentration) was added to the protein sample, which was stored at −80 °C.

For p53-TMGST purification, the same general procedure was used with some modifications. Protein expression was induced overnight with 1 mM IPTG in the presence of 0.1 mM ZnS at 16 °C. The purification protocol was as above, except that positive fractions from the Ni-NTA agarose column were dialysed (2 h, 4 °C) with A-GST buffer (150 mM KCl, 2 mM KH_2_PO, 4.5% (w/w) glycerol, 2 mM DTT, 10 mM Na_2_HPO_4_ pH 7.4) and loaded onto a GSTrap column (GE Healthcare). Protein was eluted with B-GST buffer (200 mM KCl, 5% w/w glycerol, 5 mM DTT, 50 mM reduced glutathione, 50 mM Hepes pH 7.4) and fractions analysed by SDS-PAGE. Fractions containing the protein were pooled and concentrated to 5 mL in a 50 K cut-off Amicon filter and loaded onto a HiLoad 16/60 Superdex 200, as explained above and used throughout.

For purification of CHIP, UbcH5a, Bag1s and p53_DBD_, cells were lysed, the extracts clarified and loaded onto a Ni-NTA agarose column as above. His-tags were removed after overnight cleavage with 50 μL TEV protease (5 mg/mL) at 4 °C, and dialysed with Buffer A. After a second round of nickel affinity chromatography, fractions containing unbound protein were collected and analysed by SDS-PAGE. Positive fractions were pooled and concentrated to 5 mL using a 10 K cut-off Amicon filter, loaded onto a HiLoad 16/60 Superdex 200, and selected fractions were pooled and stored at −80 °C after supplementation with 5% glycerol.

### Ubiquitination assays

Ubiquitination assays followed the protocol of Murata and collaborators^53^ with some modifications. Briefly, chaperones (1 μM) were incubated with 500 ng substrate (p53-TMGST), 500 ng UbcH5a ubiquitin-conjugating enzyme (E2), and 50 ng human ubiquitinating enzyme E1 (Enzo Lifesciences) in 30 μL reaction buffer (50 mM KCl, 5 mM MgCl_2_ 5 mM ATP, 2 mM DTT, 20 mM Hepes pH 7.4). The amount of CHIP was optimised to reduce auto-ubiquitination species. The solution was incubated (2 h, 35 °C) and the reaction was terminated by addition of SDS-PAGE sample buffer. Samples were loaded onto NuPAGE 4–12% Bis-Tris gels (Invitrogen) and electrophoresis was carried out in MOPS SDS Running Buffer (Invitrogen) at 25 mA. SDS-PA gels were transferred to PVDF membranes (BioRad) with Mini Trans-Blot equipment (BioRad). Primary monoclonal anti-p53 antibody pAB 1801 (1/2000 dilution; Thermo Scientific) was used to identify the ubiquitinated substrate. Horseradish peroxidase-conjugated secondary anti-mouse antibody (1/5000; GE Healthcare) was used to detect the protein by ECL (BioRad). The reaction was recorded on photographic film.

### Gel filtration analysis of complexes

Protein complexes were incubated in incubation buffer (100 mM KCl, 5 mM MgCl_2_, 20 mM HEPES pH 7.4) with nucleotide (when p53-TMGST was present) at various molar ratios. The mixture was loaded onto a Superdex 200 Increase 10/300 GL column (GE Healthcare) equilibrated with the same buffer (room temperature). The protein composition of each fraction was determined by SDS-PAGE followed by Coomassie blue staining.

### Crosslinking and mass spectrometry analysis

Proteins were incubated in conditions as for gel filtration analysis (40 min, room temperature) and then loaded on Superdex 200 Increase 10/300 GL. The complex was detected by SDS-PAGE and selected fractions were crosslinked by adding fresh DTSSP or DSSO^54^ (both from Thermo Scientific; 2 mM final concentration) and incubated (30 min, room temperature). The reaction was terminated by adding Tris-HCl pH 7.4 (50 mM final concentration) for 15 min. Crosslinking efficiency was examined by SDS-PAGE in reducing and non-reducing conditions. Purified DTSSP-crosslinked complexes were precipitated using the methanol/chloroform method, and the protein pellet was dissolved in 10 µL 8 M urea, 25 mM NH_4_HCO_3_. After vigorous vortexing, the urea concentration was reduced to 2 M by addition of 30 µL 25 mM NH_4_HCO_3_, followed by addition of 100 ng proteomics-grade trypsin (Sigma-Aldrich) and digestion (5 h, 37 °C). The resulting peptide mixture was speed-vac dried and redissolved in 0.1% formic acid.

### LC-MS/MS analysis of DTSSP-crosslinked samples

The LC-MS/MS analysis was carried out using a nano-LC Ultra HPLC (Eksigent, Framingham, MA) coupled online with a 5600 triple TOF mass spectrometer (AB Sciex, Framingham, MA) through a nanospray III ion source (AB Sciex) equipped with a fused silica PicoTip emitter (10 μm × 12 cm; New Objective, Woburn, MA). The HPLC setup included an Acclaim PepMap 100 trapping column (100 μm × 2 cm, 5 μm particle size; Thermo Scientific, Waltham, MA) and an Acquity UPLC BEH C18 column (75 μm × 150 mm, 1.7 μm particle size; Waters, Milford, MA). Solvent A and B were, respectively, 0.1% formic acid and 0.1% formic acid in acetonitrile. Peptides were fractionated at a flow-rate of 0.250 mL/min at 50 °C under gradient elution conditions consisting of 2% B for 1 min, a linear increase to 30% B in 109 min, a linear increase to 40% B in 10 min, a linear increase to 90% B in 5 min and 90% B for 5 minutes. The ion source was operated in positive ionization mode at 150 °C with a potential difference of 2300 V. Each acquisition cycle included a survey scan (350–1250 m/z) of 250 ms and a maximum of 25 MS2 spectra (100–1500 m/z). For peptide identification, PeakView (v1.2, AB Sciex) or MSconvert (v3.0.6965, ProteoWizard) were used to convert raw MS/MS data to an mgf file that was searched against a custom-made database containing the sequences of the proteins in each complex. The MS/MS ion search was performed using SIM 1.3.0.3^31^ with the following settings: trypsin (fully specific) as enzyme allowing 5 missed cleavages, DSP/DTSSP as crosslinker, and MS and MS/MS tolerances of 20 ppm.

### LC-MS/MS analysis of DSSO-crosslinked samples

For DSSO samples, dried crosslinked proteins were dissolved in 6 M urea, 200 mM NH_4_HCO_3._ Samples were reduced with DTT (600 nmols, 1 h, 37 °C) and alkylated in the dark with iodoacetamide (1200 nmol, 30 min, 25 °C). The resulting protein extract was diluted 6-fold with 200 mM NH_4_HCO_3_ and digested with 20 µg trypsin (V5113; Promega; overnight, 37 °C). The peptide mix was acidified with formic acid and desalted in a MacroSpin C18 column (The Nest Group), and 97.5% of the sample was further fractionated by strong cation exchange chromatography (SCX; Empore cation-SR, 3 M) in a procedure adapted from Rappsiber and collaborators^55^. Peptides were eluted with six C_2_H_7_NO_2_ concentrations (40, 80, 120, 160, 200, 500 mM). Each fraction was desalted on a MicroSpin C18 before to LC-MS/MS analysis. Unfractionated samples (1 µg) and 30% of each SCX fraction were analysed in an Orbitrap Fusion Lumos mass spectrometer coupled to an EasyLC nano-HPLC (Proxeon). Peptides were loaded directly onto the analytical column and separated by reverse-phase chromatography on a 50-cm column (inner diameter of 75 µm) packed with 2 µm C18 particles. Solvent A was 0.1% formic acid and solvent B was 0.1% formic acid in acetonitrile. Chromatography was carried out at a flow rate of 300 nl/min starting with 5% buffer B and gradually increasing to 30% buffer B in 52 min and to 40% buffer B in 8 min. After each analysis, the column was washed for 10 min with 5% buffer A and 95% buffer B.

The mass spectrometer was operated in data-dependent mode and full MS scans were acquired in the Orbitrap mass analyzer at a resolution of 60,000 over a mass range of m/z 375–1500. Auto gain control (AGC) was set to 4E5 and dynamic exclusion to 30 seconds. In each cycle, the most intense ions with charges 3 to 8 above a threshold ion count of 1E4 were selected for CID fragmentation at normalized collision energy of 25% with detection in the Orbitrap. Subsequently, mass-difference-dependent MS3 acquisitions were triggered if a unique mass difference of 31.9721 Da was observed in the CID-MS2 spectrum. In the MS3 the HCD normalised collision energy was set at 30% and the detection was carried out in the linear ion trap. All data were acquired with Xcalibur software v3.0.63. The mass spectrometer was controlled with Xcalibur software (v3.0.63; Thermo Fisher). Proteome Discoverer software suite (v2.2, Thermo Fisher Scientific), Mascot search engine (v2.5, Matrix Science)^56^; and XLinkx v2.2^57,58^; were used for peptide identification. Samples were searched against a SwissProt database containing entries corresponding to human (number entries = 20,810), a list of common contaminants and all the corresponding decoy entries. Trypsin was chosen as the enzyme and a maximum of three miscleavages were allowed. Carbamidomethylation (C) was set as a fixed modification, whereas oxidation (M), acetylation (N-terminal) and DSSO modifications (K) (monolink ammonia and water quench, intra-peptide and inter-peptide crosslink) were used as variable modifications. Searches were performed using a peptide tolerance of 7 ppm, and a product ion tolerance of 20 mmu. Crosslinked peptides were filtered at a FDR < 1% and an XlinkX score > 80 and all the MS2 spectra of the resulting peptides were manually revised. The mass spectrometry proteomics data have been deposited to the ProteomeXchange Consortium via the PRIDE^59^ partner repository with the dataset identifiers PXD009756 and PXD009777”.

### Stabilisation and isolation of complexes using GraFix

Complexes were stabilised and isolated using the GraFix technique^28^. Complexes were formed in the same molar conditions and incubated in the same buffer as for gel filtration analysis. After incubation (60 min, room temperature), samples were deposited on a 10–30% glycerol gradient in incubation buffer (100 mM KCl, 5 mM MgCl_2_, 20 mM Hepes pH 7.4). Continuous gradients were formed using a Gradient Master device (BioComp). The heavy solution contained 0.15% glutaraldehyde; as a control, a glutaraldehyde-free gradient was run in parallel. Samples were ultracentrifuged (132,000 xg, 16 hr, 4 °C) using an SW55 Ti rotor in a Beckman XL-90 ultracentrifuge. Fractions were collected manually from the top of the tube and analysed by SDS-PAGE in 10% or 6% polyacrylamide gels for controls and crosslinked samples, respectively. Gels were silver-stained using standard protocols and positive fractions evaluated by EM.

### Electron microscopy

#### Sample preparation and data collection

Protein complexes were analysed by transmission electron microscopy (TEM) using the negative stain technique. Aliquots (5 μL) of the protein sample at appropriate concentration were adsorbed for 5 min onto glow-discharged, carbon-coated copper/rhodium grids (Quantifoil). Grids were washed and incubated (1 min) with 5 μL of 2% (w/v) uranyl acetate. Samples were examined on an 80 kV JEOL JEM1010 microscope; images were acquired following low electron dose protocols. Images were recorded at 52,000× magnification, with 2–4 µm underfocus values, using a 4K × 4K digital TemCam-F416 camera (TVIPS) with 15.6 μm pixel size (3 Å/pixel sampling ratio).

For cryoelectron microscopy (cryoEM), selected fractions from GraFix experiments were concentrated in an Amicon Ultra 0.5 mL (50 kDa cut-off; 4,000 g, 5 min, 4 °C), fresh incubation buffer was added to remove glycerol, followed by four additional centrifugation rounds. Samples were analysed by negative staining to confirm they were unaffected by glycerol removal. Sample aliquots (3 μL) were adsorbed onto glow-discharged, R 1.2/1.3 copper/rhodium 300 mesh grids (Quantifoil). Grids were vitrified semi-automatically using a Vitrobot Mark IV (FEI; 22 °C, 100% humidity) and single-blotted (2 sec, 0 force) before plunging into liquid ethane pre-cooled with liquid nitrogen. Images of the Hsp70:p53-TMGST:CHIP complex were taken at the Diamond Light Source facility on an FEI Titan Krios electron microscope operating at 300 kV with 81,000× nominal magnification. Movies were recorded (a total of 1600) with a K2 Summit detector post-GIF (Gatan) with super resolution mode (0.9 Å/pix); defocus varied from 1.6 to 3.5 μm. Each movie was separated into 30 frames (0.5 s each; total exposure 15 s) with a dose rate of 6.21 e^−^/Å/s for a total dose of 36 e^−^/Å. Images of the Hsp90:p53-TMGST:CHIP complex (total 1200) were taken on a Talos Arctica electron microscope equipped with a Falcon III direct detector (FEI) operated at 200 kV with a 72,000× nominal magnification. Movies were recorded in counting mode, 81 s exposure distributed in 90 fractions (38 frames/fraction, 0.87 s/fraction) and a total dose of 30 e^−^/Å^2^ (1.31 e^−^/Å^2^/fraction). Defocus ranged from −1.6 to −3.5 μm. The sampling ratio was 1.42 Å/pixel.

#### 3D reconstruction

Images were processed using Scipion Software^60^. For movies, motion induced in the sample by the electron beam was corrected using MotionCorr2^61^. The contrast transfer function (CTF) was determined by CTFfind4^62^. Particles were selected from the images manually or, when possible, automatically using Xmipp autopicking software^29^. Particles were extracted from the downsampled images with Xmipp^63^. To reduce computational demand and to increase signal/noise ratio, images from the JEOL JEM1010 were downsampled by 2, rendering 5.95 Å/pixel. For cryoEM images, particles were extracted with a downsampling factor of 3, whereas for the last refinements, particles were used at the original sampling ratio to improve the final resolution. Particles were classified two-dimensionally using Relion^30^. Several 2D analyses were performed to discard low quality particles and false positives derived from automatic picking procedures. Initial models for all processes were obtained with the EMAN2 initial model^64^, using the averages obtained from the 2D classification. For each project, initial models were refined with the selected set of particles from the 2D classification using Relion. Those that showed correct convergence were used for the next step. Once a correct model was obtained, the particles were used for 3D classification with Relion. The highest resolution seeds were selected for the final refinement of selected volumes in Relion. The models were validated at two levels; for the 2D level, various orientations of the model obtained in the reference-free 2D classifications were compared with theoretical projections and reference-based classes obtained during refinement. The 3D refinement procedure was performed simultaneously and independently, using different initial models with the same data set. When a similar final model was obtained, it suggested that the 3D reconstruction accurately represented the structural information from the single-particle data. Resolution of the final 3D models was estimated based on the Fourier shell correlation (FSC) with spatial frequency at 0.3 correlation^65^. Visualization of the 3D models and docking of the atomic structures into EM volumes was performed manually using USCF Chimera^66^. The atomic structures used throughout the study were from the Protein Data Bank (http://www.rcsb.org/pdb).

### Accession codes

The 3D reconstructions of the Hsp70:p53-TMGST:CHIP and Hsp90:p53-TMGST:CHIP complexes generated by cryoEM have been deposited in the EMDB (code EMD4293 and EMD4294, respectively).

## Supplementary information

Supplementary information
