# Supplementary material for: The cochaperone CHIP marks Hsp70- and Hsp90-bound substrates for degradation through a very flexible mechanism
Source: Sci Rep. 2019 Mar 25;9:5102. doi: 10.1038/s41598-019-41060-0 (PMC6433865; doi:10.1038/s41598-019-41060-0)
Supplement: Supplementary file 1 — Supplementary information [file 41598_2019_41060_MOESM1_ESM.pdf]

The cochaperone CHIP marks Hsp70- and Hsp90-bound substrates for degradation through a very flexible mechanism

[1]Lucía Quintana-Gallardo, [1]Jaime Martín-Benito, [1]Miguel Marcilla, [2,3]Guadalupe Espadas, [2,3]Eduard Sabidó, [1]José María Valpuesta*

[1]Centro Nacional de Biotecnología (CNB-CSIC), Darwin 3, 28049 Madrid, Spain. [2]Proteomics Unit, Centre de Regulació Genòmica (CRG), Barcelona Institute of Science and Technology (BIST), Barcelona, Spain. [3]Proteomics Unit, Universitat Pompeu Fabra, Barcelona, Spain.

Correspondence should be addressed to J.M. Valpuesta (jmv@cnb.csic.es).

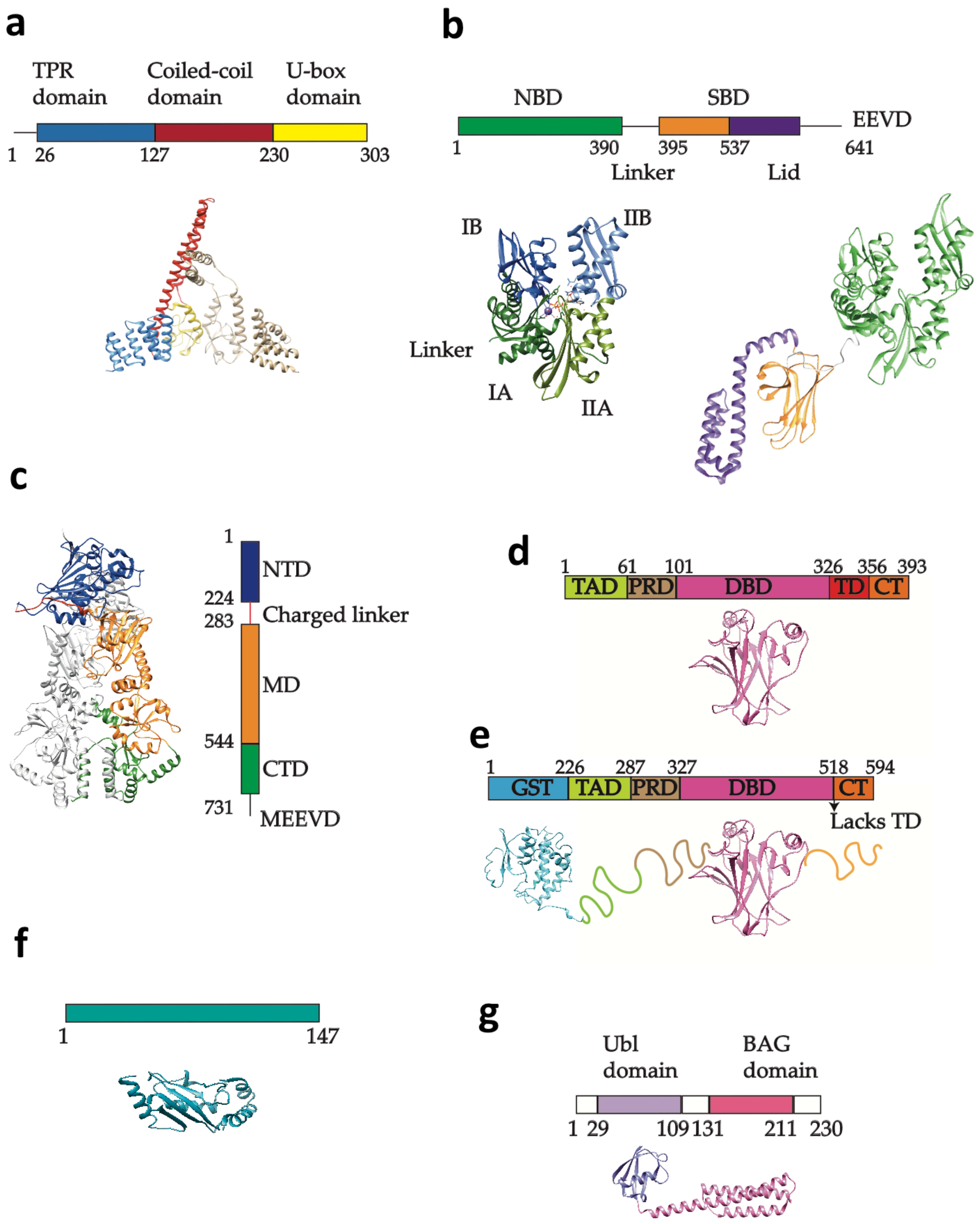

Supplementary Figure 1. Sequence and atomic structure of the chaperones, cochaperones and substrate used. a) The CHIP sequence with its domains. The TPR domain (blue) that interacts with the chaperones is located in the N-terminal domain. The coiled-coil domain (red) participates in protein dimerization. The U-box domain (yellow) has E3 ligase activity and is also responsible for dimerization. The crystal structure of murine CHIP (pdb 2c2l; (Zhang et al., 2005) shows an asymmetric dimer whose three domains are indicated in the colour scheme. **b)** Hsp70 structure. The NBD (green) is followed by a linker that connects with the C-terminal, substrate-binding domain (SBD). The SBD can be divided

into the α (orange) and the β subdomains (purple), and ends with the EEVD motif, which is recognized by some cochaperones. The crystal structure of the human $Hsp70_{NBD}$ with no nucleotide (pdb 3atv), with subdomains in (IB, dark blue; IIB, light blue; IA, dark green; IIA, light green) and the same domain with the $Hsp70_{SBD}$ (pdb 4po2**)** connected by the linker. **c)** Left, scheme showing the Hsp90 sequence and its domains. The NTD (blue) bears the ATPase domain and the binding site for the inhibitor geldanamycin. The charged linker (red) connects the NTD with the MD (orange), where most substrates bind. The CTD (green) has the dimerization domain and the MEEVD motif for TPR-containing cochaperone binding. Right, the atomic structure of human Hsp90 (pdb 5fwl (Verba et al., 2016)) in the ATP conformation. One monomer is shown in beige; the other follows the colour scheme at left. **d)** The p53 domains are shown (N-terminal transactivation domain, TAD; green; proline-rich domain, PRD; brown; DNA-binding domain, DBD; pink; tetramerization domain, TD; red; C-terminal domain, CT; orange). Bottom, the atomic structure of the human DBD (pink)(pdb 2ocj; Wang, Rosengarth, & Luecke, 2007). **e)** Sequence and atomic structure of the p53-TMGST chimeric protein. Top, the p53-TMGST sequence. In this chimeric protein, the p53 tetramerization domain was removed and the Glutathione S transferase (GST) (cian) (pdb 1m99) added to the N-terminal domain. Bottom, the atomic structure of human $p53_{DBD}$ (pink), the representation of the disordered domains and crystal structure of GST fusion protein (cian). **f)** Sequence and atomic structure of the E2 ubiquitin conjugating enzyme UbcH5a (pdb 2oxq). **g)** Sequence and atomic structure of Bag1s from *Arabidopsis thaliana* (pdb 4hwi (Fang et al., 2013)), which contains the Bag (pink) and Ubl (purple) domains.

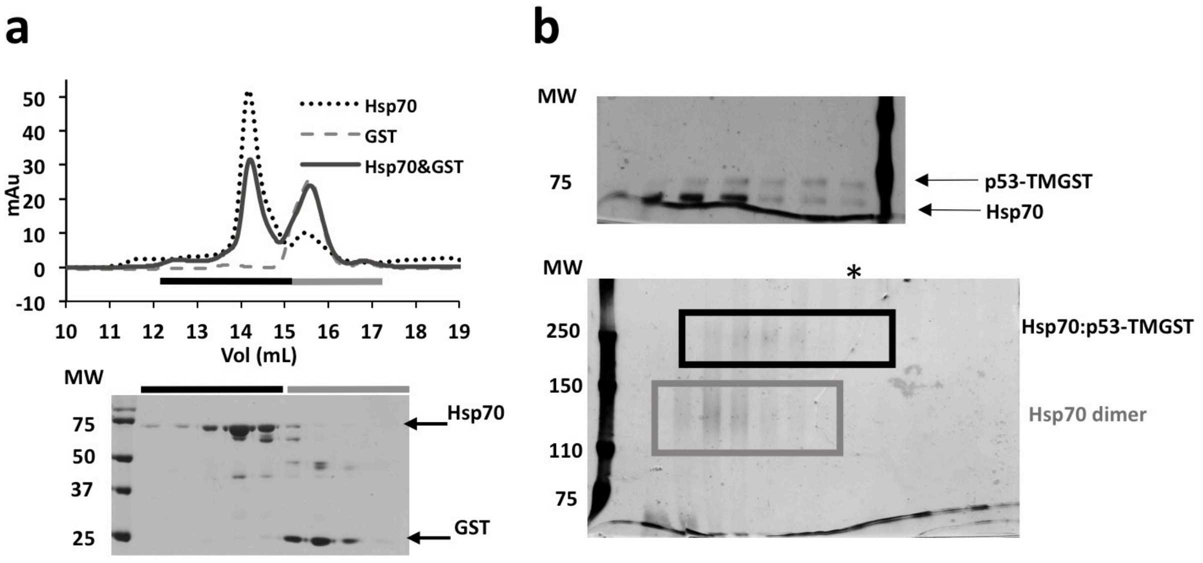

c

Hsp70		p53-TMGST		Distance (A)
aa	Fragment	aa	Fragment	
275/276/277	TL**SSS**TQASLEIDSLFEGIDFYTSITR	486	RPILTIITLED**S**SGNLLGRNSFEVHV CACPGRDR	22
40/56	TTPSYVAFTDTERLIGDAA**K**NQVALNP QNTVFDA**K**	409/411	C**S**D**S**DGLAPPQHLIR	36
418/423	RN**S**TIPT**K**QTQIFTTYSDNQPGVLIQVY EGER	107/112/113	YGV**S**RIAY**SK**DFETLK	36
56	LIGDAAKNQVALNPQNTVFDA**K**	535/540/541/545	AL**S**NNTS**SS**PQP**K**K	
85/88	FGDPVVQ**S**DM**K**HWPFQVINDGDKPK	573/576/577	GQST**S**RH**KK**LMFK	
325/328	DA**K**LD**K**AQIHDLVLVGGSTR	566/657/571/573	**SK**KGQ**S**T**S**RHK	
325	DA**K**LD**K**AQIHDLVLVGGSTR	562/565/566/568	AHS**S**HL**KS**K**K**GQSTSR	

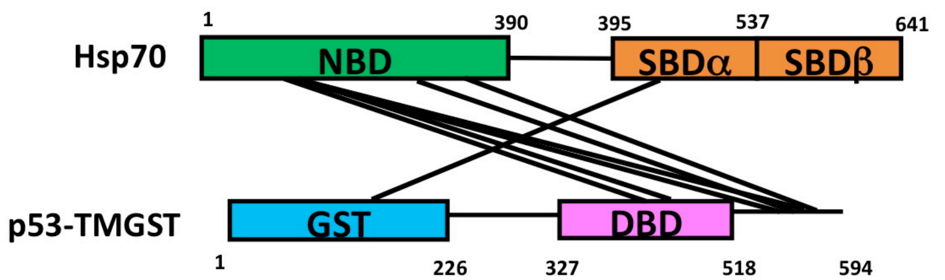

Supplementary Figure 2. Biochemical analysis of the Hsp70:p53-TMGST interaction. a) GST does not interact with Hsp70. Size-exclusion profile of the putative Hsp70:GST complex, and of each individual protein as controls. The SDS, Coomassie blue stained gel shows the complex fractions. The gel shows only Hsp70 in the first peak; the second peak, which overlaps with that of control GST in the profile, shows only GST in the gel. **b)** GraFix

Purification of the Hsp70:p53-TMGST complex. Glycerol gradient fractions without (top) or with (bottom) glutaraldehyde. Increasing glycerol concentration from left to right. The gels were silver stained. The asterisk indicates the fraction used for EM. **c)** XL-MS analysis of the Hsp70:p53-TMGST complex. Top, table showing the highest DTSSP crosslinking score obtained by MS; amino acids in bold participate in the peptide crosslink. Distances described are only those between structured domains. Bottom, scheme of data at the top.

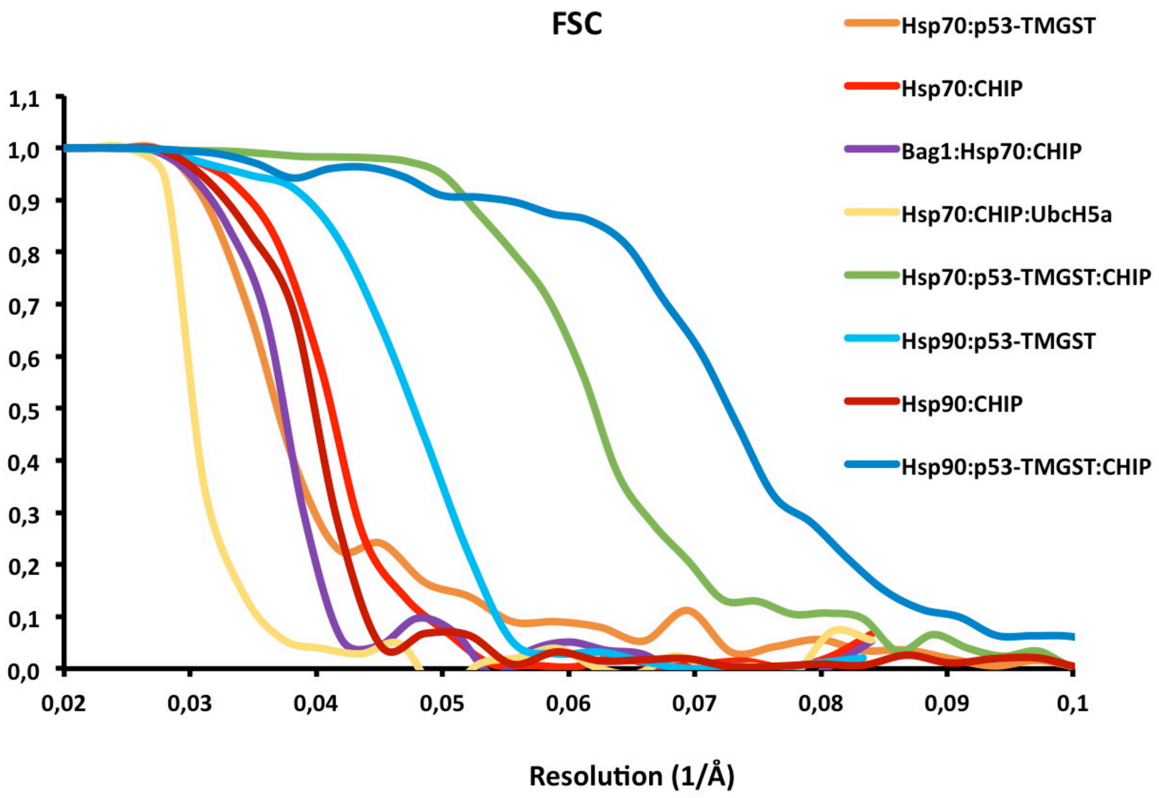

Supplementary Figure 3. Fourier shell correlation (FSC) of the 3D reconstructions described in this work. Resolution of the final 3D models was estimated based on the Fourier shell correlation (FSC) with spatial frequency at 0.3 correlation[65]

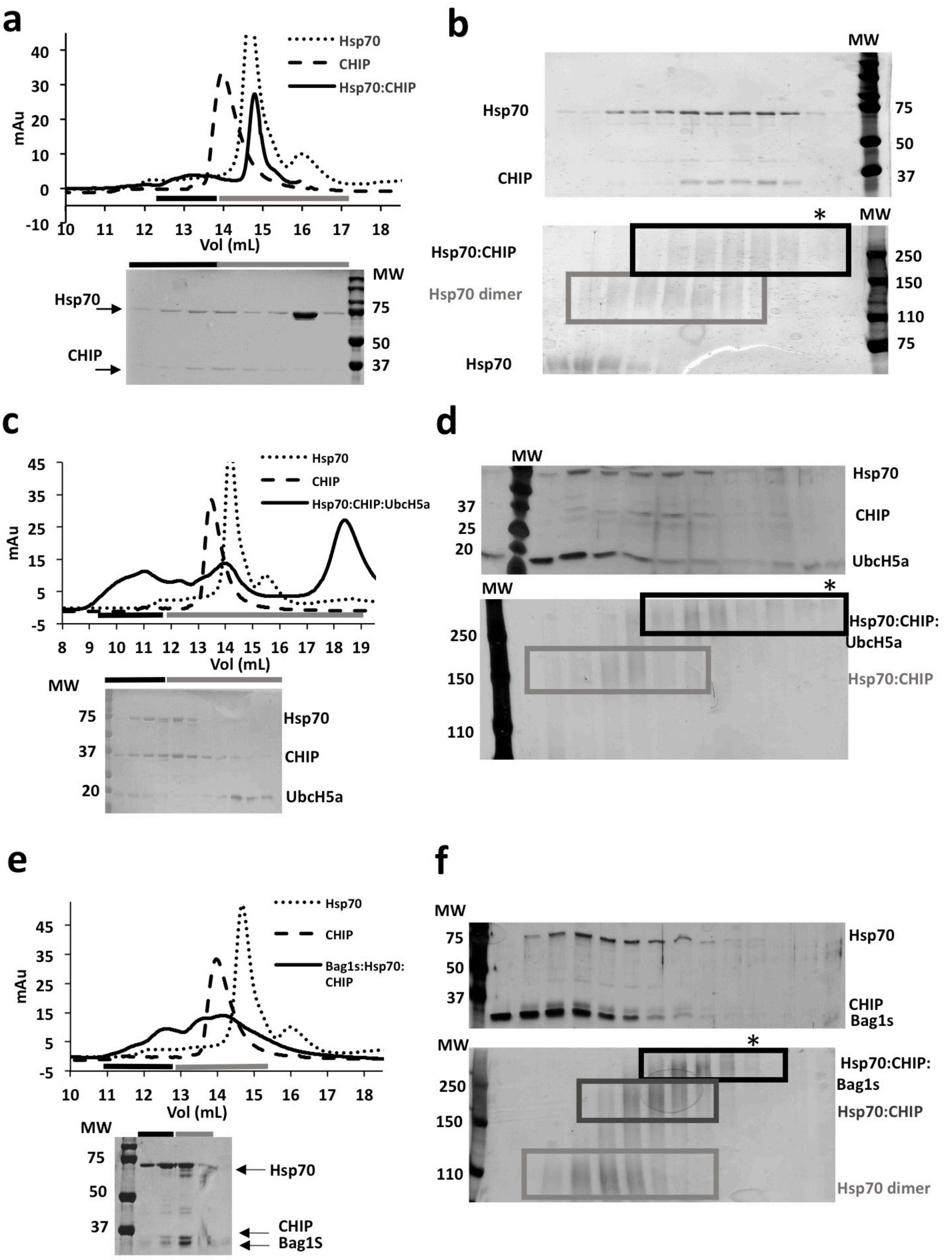

Supplementary Figure 4. Isolation of the Hsp70:CHIP-based complexes. a) Size exclusion profile of the Hsp70:CHIP complex and of the Hsp70 and CHIP controls. **b)** GraFix purification of the Hsp70:CHIP complex. Glycerol gradient fractions without (top) or with (bottom) glutaraldehyde. Increasing glycerol concentration from left to right. The gels were silver stained. **c)** and **d)** as in a) and b), with the Hp70:CHIP:UbcH5a complex. **e)** and

f), as in a) and b), with the Hsp70:CHIP:Bag1s complex. The asterisks in b), d) and f) mark the lanes used for EM.

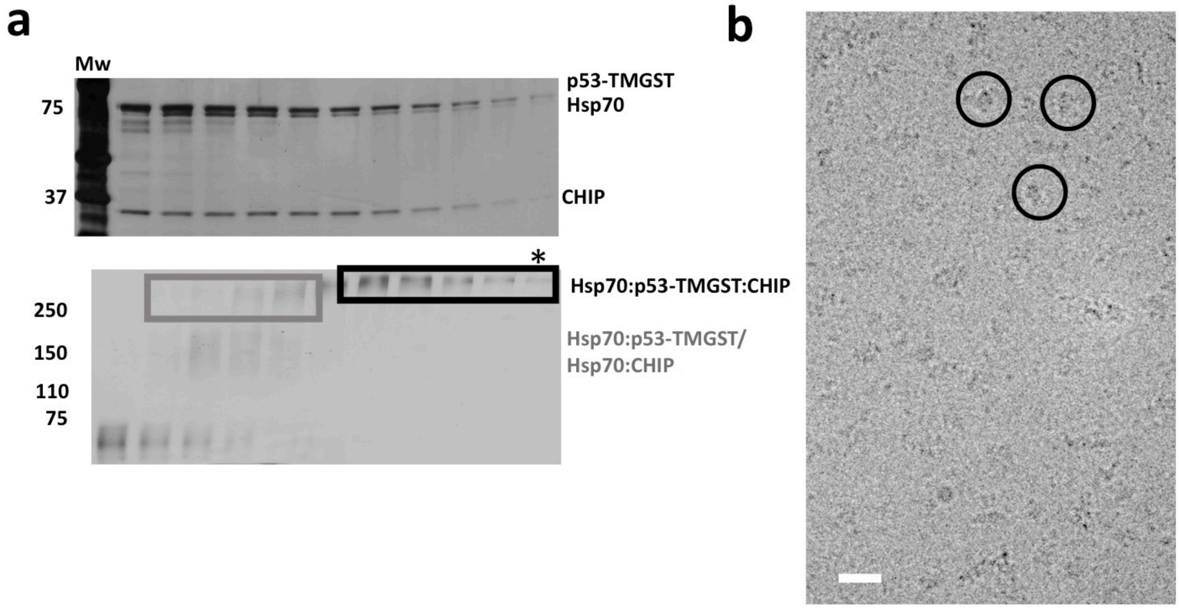

c

Hsp70		p53-TMGST		Distance (A)
aa position	Fragment	aa position	Fragment	
77/85/88	**K**FGDPVVQ**S**DM**K**HWPFQVI NDGDKPK	44/45	N**KK**FELGLEFPNLPYYIDGDVK	30
362/384	**S**INPDEAVAYGAAVQAAILMG D**K**SENVQDLLLLDVAPLSLGLE TAGGVMTALIK	562/565	RALSNNTSSSPQPKKKPLDGEYPGGSRAHS**S** HL**K**SK	
**Hsp70**		**CHIP**		
597/608	R**K**ELEQVCNPII**S**GLYQGAGG PGPGGFGAQGPK	19/22/23/30	LGAGGG**S**PE**KS**PSAQEL**K**EQGNRLFVGRK	32
**p53TMGST**		**CHIP**		
539/535/ 540/545	AL**S**NNT**SS**SPQP**K**K	22/23/25	LGAGGGSPE**KS**P**S**AQELK	

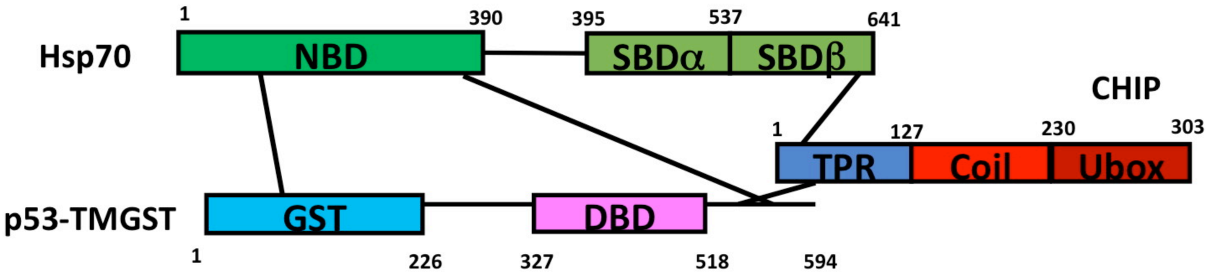

Supplementary Figure 5. Biochemical analysis of the Hsp70:p53-TMGST:CHIP interaction. a) GraFix purification of the Hsp70:p53-TMGST:CHIP complex. Glycerol gradient fractions without (top) or with (bottom) glutaraldehyde. Increasing glycerol concentration from left to right. The asterisk indicates the fraction used for EM. **b)** Image of a vitrified specimen of the Hsp70:p53-TMGST:CHIP complex. **c)** XL-MS analysis of the Hsp70:p53-TMGST:CHIP complex. Top, table showing the highest DTSSP crosslinking

score obtained by MS; amino acids in bold participate in the peptide crosslink. Distances described are only those between structured domains. Bottom, scheme of data at the top.

a

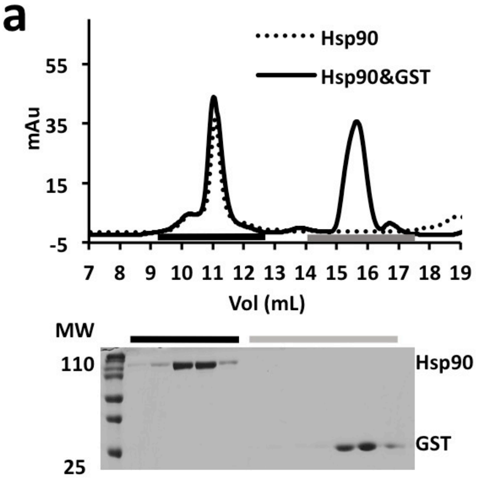

b

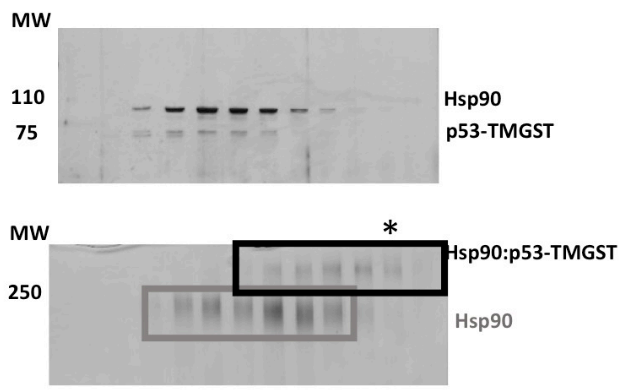

c

Hsp90		p53-TMGST		Distance (A)
aa position	Fragment	aa position	Fragment	
658	SV**K**DLVILLYETALLSSGFSLEDPQTHANR	43	DEGD*K*WRNK	12
362	NNI**K**LYVRR	365	MFCQLA**K**TCPVQLWVDSTPPPGTR	27
660	SV**K**DLVILLYETALLSSGFSLEDPQTHANR	547	PLDGEYPGG**S**R	

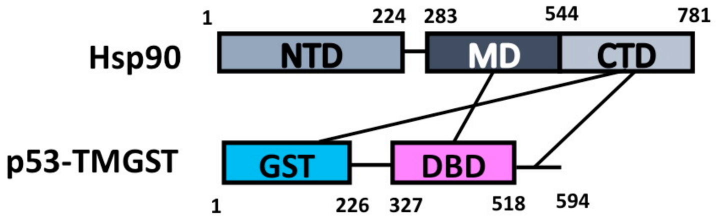

d

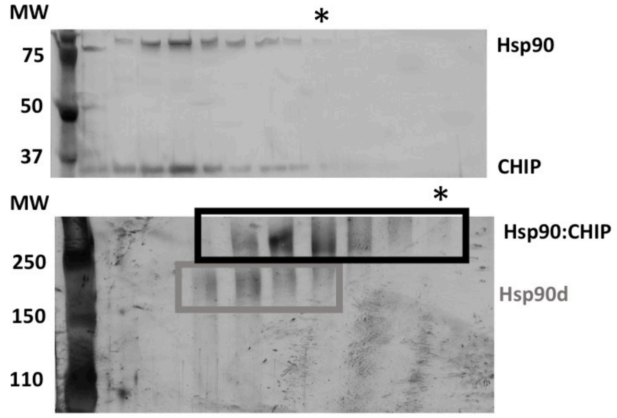

Supplementary Figure 6. Biochemical analysis of the Hsp90:p53-TMGST interaction. a) GST does not interact with Hsp90. Size-exclusion profile of the putative Hsp90:GST complex, and of each individual protein as controls. **b)** GraFix purification of the Hsp90:p53-TMGST complex. Glycerol gradient fractions without (top) or with (bottom) glutaraldehyde. Increasing glycerol concentration from left to right. **c)** XL-MS analysis of the

Hsp90:p53-TMGST complex. Top, table showing the highest DTSSP crosslinking score obtained by MS; amino acids in bold participate in the peptide crosslink. Distances described are only those between structured domains. Bottom, scheme of the crosslinks shown at top. **d)** GraFix purification of the Hsp90:CHIP complex. Glycerol gradient fractions without (top) or with (bottom) glutaraldehyde. Increasing glycerol concentration from left to right. The asterisks in b) and d) mark the lanes used for EM.

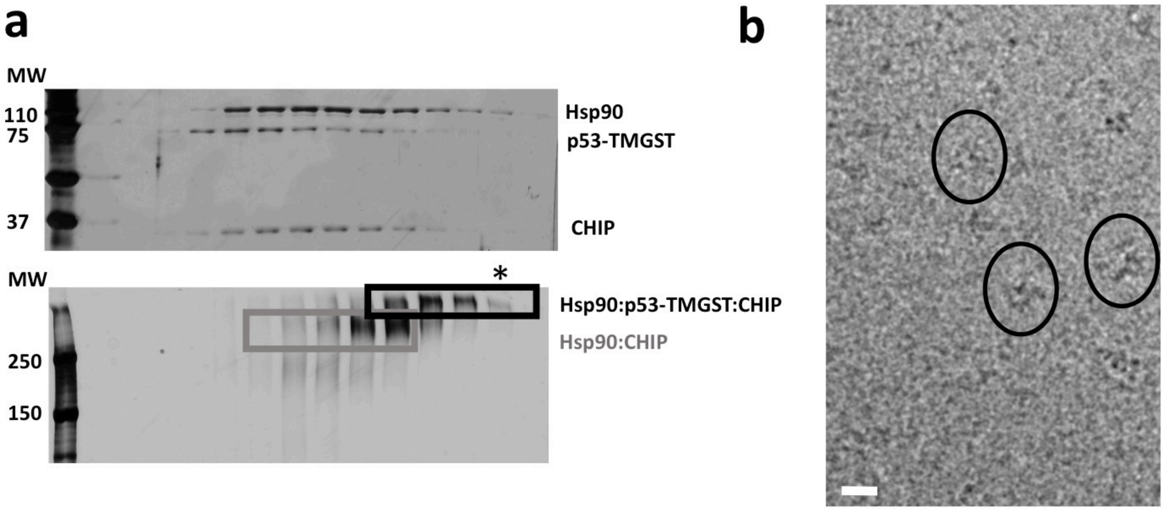

c

Hsp90		p53TMGST		Distance (A)
aa position	Fragment	aa position	Fragment	
678/680	**S**V**K**DLVILLYETALLSSGFSLEDPQTHANR	40	DEGD**K**WRNK	32
678/680	**S**V**K**DLVILLYETALLSSGFSLEDPQTHANR	557	PLDGEYPGG**S**R	
**Hsp90**		**CHIP**		**Distance (A)**
aa position	Fragment	aa position	Fragment	
559	A**K**FENLCK	22	LGAGGGSPE**K**SPSAQELK	14
607	IM**K**AQALR	22	LGAGGGSPE**K**SPSAQELK	33
607	IM**K**AQALR	7	EE**K**EGGAR	33
607	IM**K**AQALR	30	SPSAQEL**K**EQGNR	38
354	NNI**K**LYVR	30	SPSAQEL**K**EQGNR	40
69	YESLTDPSKLDSG**K**ELK	41	**K**YPEAAACYGR	32
69	LDSG**K**ELK	22	LGAGGGSPE**K**SPSAQELK	24

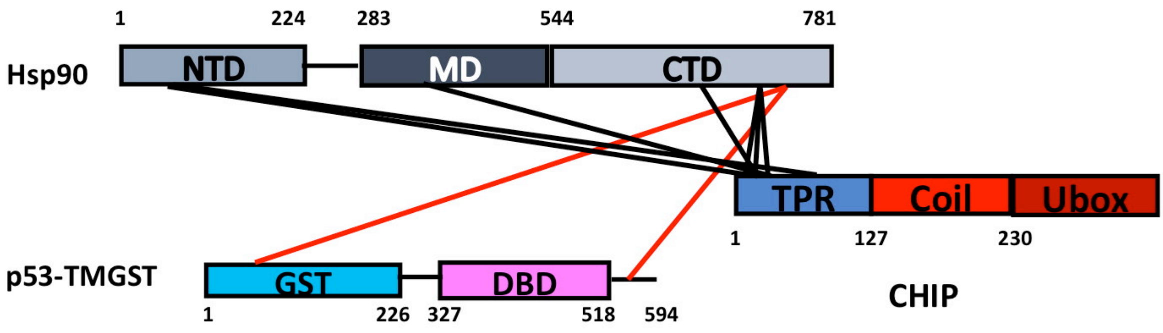

Supplementary Figure 7. Biochemical analysis of the Hsp90:p53-TMGST:CHIP interaction. a) GraFix purification of the Hsp90:p53-TMGST:CHIP complex. Glycerol gradient fractions without (top) or with (bottom) glutaraldehyde. Increasing glycerol concentration from left to right. The asterisk marks the lane used for EM. **b)** Image of a

vitrified specimen of the Hsp90:p53-TMGST:CHIP complex. **c)** XL-MS analysis of the Hsp90:p53-TMGST complex:CHIP complex. Top, table showing the highest crosslinking score obtained by MS; amino acids in bold participate in the peptide crosslink. Distances described are only those between structured domains. Bottom, scheme of data shown at top. The crosslinks coloured in red are from experiments with DTSSP and in those in black from experiments with DSSO.

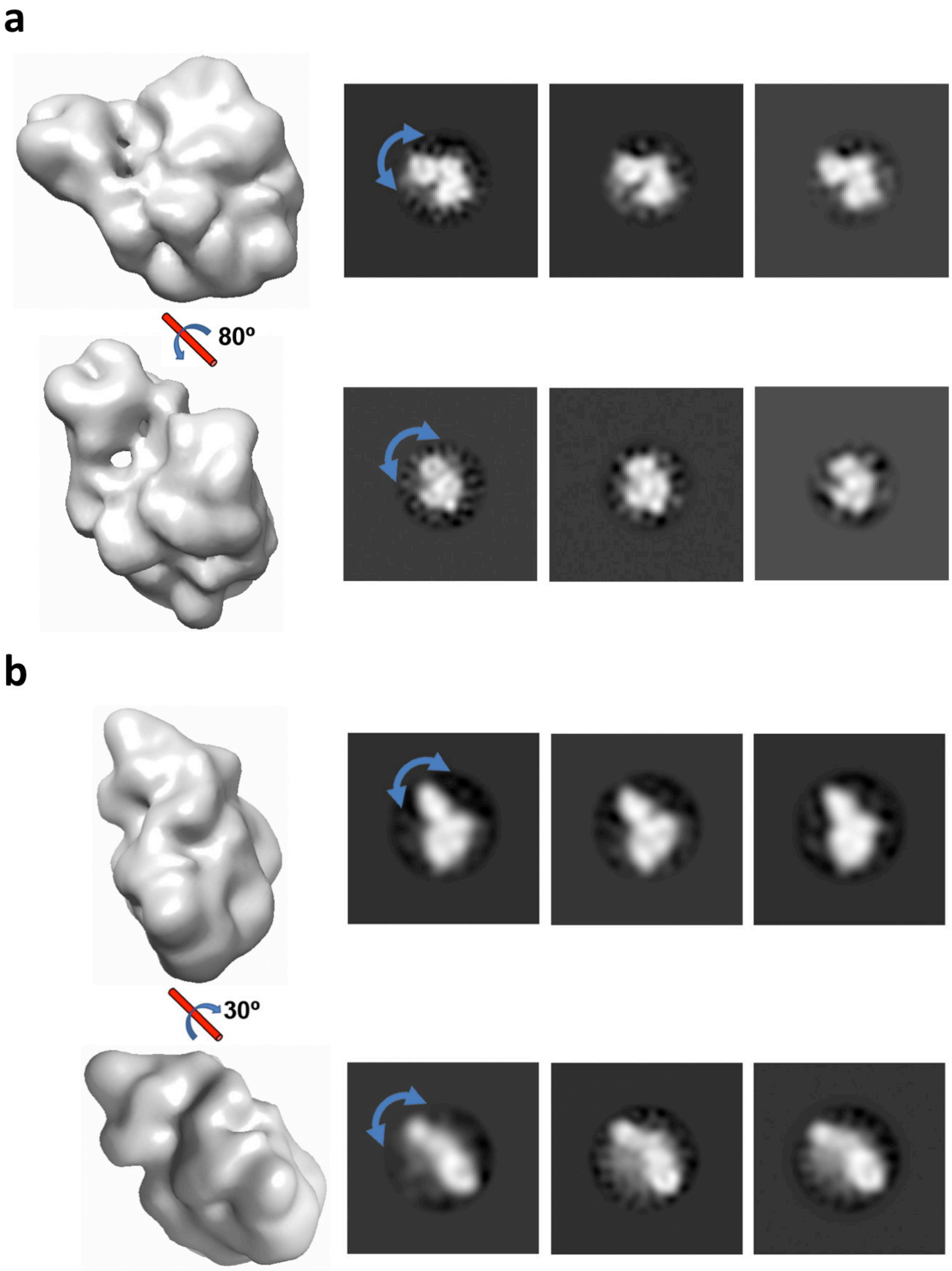

Supplementary Figure 8. Flexibility of the chaperone/chaperone interaction. a) Images showing part of the flexibility of the Hsp70:p53-TMGST:CHIP complex. Left column, two views of the Hsp70:p53-TMGST:CHIP 3D reconstruction. Right, three different 2D classes obtained after classification with Relion, associated with the corresponding view of the complex on the left. **b)** as in a), for the Hsp90:p53-TMGST:CHIP complex. For all the cases, the two-headed arrows intend to show rocking of the smaller mass in relation to the larger one.
